# Changing Perceptions of Ornamental Plants in Urban Yangon, Myanmar

**DOI:** 10.3390/plants14040552

**Published:** 2025-02-11

**Authors:** Aung Si, Aung Kyawphyo

**Affiliations:** 1 Institute for Linguistics, University of Cologne, 50923 Koln, Germany; 2 Independent Researcher, Yangon, Myanmar; aungkp@gmail.com

**Keywords:** ornamental plants, nurseries, orchids, gardening, horticultural trade, Burmese, nomenclature, ethnobiological classification

## Abstract

Gardening is a popular pastime for people from all walks of life in Yangon, the most populous city of Myanmar and its former capital. The cultivation of ornamental plants has continued uninterrupted despite decades of social and political crises that have plagued the country, but there are indications that people’s tastes have changed considerably. These changing tastes are documented here through interviews of nursery owners and amateur gardeners from Yangon. This study also investigates the development of naming patterns in Burmese, in particular the names coined for recently introduced plants. A total of 176 older and 156 newer (introduced over the last two decades) ornamental plants grown in Yangon were documented; of the former category, 75% were still available in nurseries, whereas the rest were no longer popular. The newer plants had significantly fewer unanalysable names than the older plants, suggesting a modern preference for descriptive/allusive labels. This also applies to native, wild-harvested orchid species. Many of the newer, introduced orchid hybrids have not been given Burmese names, but are only referred to by shorthand labels like *dendro* and *vanda*. This study provides a first, linguistically informed ethnobiological report of ornamental plants in Myanmar.

## 1. Introduction

Apart from the consumption of fruits and vegetables as food, gardening is perhaps the only activity through which urban dwellers come into regular contact with plant life. Gardening has been found to impart numerous beneficial health outcomes to the people who practice it [1]. This was particularly evident during the COVID-19 lockdowns around the world, when gardening was seen by many as a way of reducing stress, improving mental health, and maintaining general well-being [2,3,4]. On the other hand, the booming trade in ornamental plants and an ever-growing desire for showy, exotic plants among hobby gardeners has led to concerns, among conservationists, about biological invasions and subsequent negative ecological outcomes [5].

Changing fashions are a fact of urban life, and the domain of gardening is not immune to this phenomenon [6]. Demand for new and exotic plants, such as those from South and Central America, appears to be high in some southeast Asian countries [7], and the search for new ornamentals seems to be proceeding unabated in many parts of the world [8,9,10]. Gardening has always been popular in Yangon, the erstwhile capital and most populous city of Myanmar. The tropical monsoon climate allows a great variety plants to be cultivated year round, and people fortunate enough to have a garden attached to their house usually grow numerous ornamental plants—either in containers or in the ground—along with a number of edible species, such as tomato (*Solanum lycopersicum*), chili (*Capsicum annuum*), bottle gourd (*Lagenaria vulgaris*), lemon basil (*Ocimum* x *africanum*), mango (*Mangifera indica*), lime (*Citrus* x *aurantiifolia*), papaya (*Carica papaya*) and drumstick (*Moringa oleifera*). Even in the crowded apartment blocks of the old colonial city centre and elsewhere in the city, it is not uncommon to see small balconies full of orchids and other ornamentals. In the last two decades, the normally isolated country witnessed an unprecedented political and economic opening, which was accompanied by a great many changes in gardening fashions and practices. This opening proved to be short-lived, and the country has once again been plunged into violence and instability, but the new gardening trends seem to have been adopted enthusiastically by locals. This paper is a first attempt to document some of these changing fashions; in particular, it contrasts the plants cultivated by hobby gardeners in the 1980s and 1990s (i.e., prior to the economic opening mentioned above) with those available nowadays in nurseries located in Yangon.

This paper takes a linguistic approach to analysing the names given to ornamental plants, both old and new. As many of the plants available nowadays are likely to be exotic species, a key question is how these introduced plants are named by local plant sellers and gardeners, and whether these names differ qualitatively from those of older, more naturalised species. The paper will also briefly consider how Burmese ornamental plant names fare with regard to the purported ‘universals’ of ethnobiological classification [11]. Chief among these is the strong prediction that folk generics (e.g., oak, pine) should map onto biological genera (*Quercus*, *Pinus*), as the generic level reflects the principal cognitive divisions in nature. An in-depth investigation of this issue is beyond the scope of this paper, but a preliminary analysis will be carried out to test this claim with the available Burmese plant names.

In summary, the aims of this study are as follows:

1. List the ornamental plants traditionally grown by hobby gardeners in Yangon, Myanmar, in the 1980s and 1990s.
2. Document the new ornamental plant species that have become available to hobby gardeners in Yangon over the last two decades.
3. Investigate any patterns in the naming of ornamental plants, with a focus on how recent arrivals are incorporated into the Burmese lexicon.

## 2. Results

A total of 331 common plant names were documented through interviews and web searches, of which 175 represent the more traditional ornamental plants already present in household gardens in the 1980s and 1990s (henceforth, ‘older’, Table S1 in Supplementary Materials), and 156 represent modern varieties, which have appeared in the horticultural market in the last 10 years (henceforth, ‘newer’; Tables S2 and S3). Note that in general, ferns, palms, grasses, bromeliads and some bamboos have been omitted from the table, due to the difficulties inherent in determining precise botanical identifications for these taxa. Ferns are, in fact, new arrivals to the Myanmar gardening scene, as is evidenced by the fact that they are collectively labelled by the English name *fern*. Orchids are dealt with separately (Table S4). Some other plants have been omitted, because the interviews were carried out towards the end of the monsoons (October), and many angiosperms had yet to flower at that time. Tables S1 and S2 also do not contain the decorative cut flower varieties (such as lilies, gladiolus, large *Chrysanthemum* and *Dianthus* varieties, tuberose, tulips, etc.) that are grown in more temperate climates in northern and central Myanmar (see [12]) and are sold at flower markets in Yangon.

The fact that the ethnotaxa listed in Table S1 are older does not mean that they are no longer sold in plant nurseries nowadays. Indeed, 127 different ornamentals (from a total of 175, or 70%) are still available to hobby gardeners. Still popular are plants like croton (*Codiaeum variegatum*), gardenia (*Gardenia jasminoides*), frangipani (*Plumeria* spp.), champak (*Magnolia champaca*) and hydrangea (*Hydrangea* sp.). The remaining 27% consists of a number of species that were once commonplace in home gardens but are now rarely sold at plant nurseries. These include plants such as Madagascar periwinkle (*Catharanthus roseus*), coleus (*Coleus scutellarioides*), cockscombs (*Celosia argentea*), oyster plant (*Tradescantia spathacea*), cathedral bells (*Kalanchoe* spp.), crown of thorns (*Euphorbia milii*), yellow shrimp plant (*Pachystachys lutea*) and crepe myrtles (*Lagerstroemia* spp.) While it is not impossible to find these plants in Yangon, it has nevertheless become very difficult.

Some species of ornamentals appear in both Tables S1 and S2. This is because new varieties and cultivars of older plants have appeared on the horticultural market, sometimes eclipsing the popularity of the latter. This is particularly true of leafy ornamentals, as highly variegated variants of species such as *Dieffenbachia*, *Aglaonema*, *Goeppertia* and *Syngonium* have replaced their plainer counterparts in Yangon nurseries. Among the flowering plants, dwarf varieties with multicoloured flowers (as with *Bougainvillea glabra*), new flower colours (e.g., *Ixora* sp.) or larger and more prolific flowers (e.g., *Combretum indicum*) now seem to be the standard cultivars available for sale.

Distribution data from the POWO database revealed a difference between the older and newer categories, in the proportions of plants listed as present (native and introduced plants combined) and not present in Myanmar. Among the older plants, 87 (48%) were listed as present, while 95 (52%) were listed as not present. For the newer plants, only 46 (30%) were listed as present, while 106 (70%) were listed as not present. The difference between the older and newer plants was statistically significant (χ^2^ = 7.73, d.f. = 1, *p* < 0.01).

### 2.1. Naming Strategies

A range of naming strategies was evident among the local names given to ornamental plants by hobby gardeners and horticulturalists. These include native Burmese names (some unanalysable and some descriptive or allusive), Burmese calques of foreign, usually English, names and foreign names, either phonologically adapted or otherwise. The newer ornamentals are also incorporated into the ethnobotanical lexicon by the same strategies, in addition to being grouped (or ‘lumped’) together with existing ethnotaxa or being labelled by their common or cultivar names. Examples are as follows:
1.Native Burmese names (analysable: descriptive/allusive)
*caun*^2^*hmi*^3^cat-tail*Acalypha hispida*
*nya*^1^*hmwe*^3^*ban*^3^night-fragrant-flower*Cestrum nocturnum*
*sɛɁ ku*^2^*ban*^3^paper-flower*Bougainvillea glabra*2. (Native) Burmese names (unanalysable)
*ngu*^1^*Cassia fistula*

*gan*^1^*gɔ*^2^*Mesua ferrea*

*d̪əbye*^2^*Syzygium* spp.
3. Calques/translations of foreign names
*le*^3^*na*^2^*yi*^2^*pan*^3^four-o’clock-flower*Mirabilis jalapa*
*neiɁban*^2^*ca*^2^heaven-lotus*Gustavia* cf. *superba*
*hsin*^2^*nəywɛɁ pein*^3^elephant-ear-taro*Alocasia macrorrhizos*4.(Phonologically incorporated) foreign names/loanwords
million hearts*Dischidia ruscifolia*

kiss me (quick)*Euphorbia milii*

black-eyed Susan*Thunbergia alata*

*sawasdee kha* [Thai greeting]*Aloysia virgata*
5. Names with one or more foreign components
*sin*^2^*dərɛ*^3^*la*^3^*ban*^3^Cinderella-flower*Ruellia simplex*
*pu*^1^*rɔ*^3^*hi*^1^*ta*^1^*gəmoun*^3^priest [Pali]-*gəmoun**Philodendron longilobatum*
*shwe*^2^*chein*^3^gold-chain*Lophanthera lactescens*6. Names indicating geographic origin
*jəpan*^2^*zəbɛ*^2^Japan-jasmine*Wrightia antidysenterica*
*yo*^3^*dəya*^3^*dəwɛ*^2^*hmain*^3^Thai-Rangoon.creeper*Combretum indicum*
*məle*^3^*sha*^3^*bədauɁ*Malaysia-*bədauɁ**Acacia auriculiformis*7. New plants lumped with existing (botanically unrelated) ethnotaxa
*su*^3^*gan*^1^*gɔ*^2^thorn-Ceylon.ironwood*Oncoba spinosa*
*ye*^2^*wa*^3^water-bamboo*Equisetum hyemale*
*sin*^2^*ga*^2^*pu*^2^*ban*^2^*da*^2^Singapore-almond*Ficus lyrata*

In addition, there seems to be at least one amusing case of a ‘Burmese’ name being coined through the misreading of a botanical name. The small South American tree *Tabebuia aurea* is called *təbe*^2^ *bu*^2^ *ta*^1^ in Yangon, but the Burmese name is unanalysable and is suspiciously similar to the genus name *Tabebuia*. One could easily imagine *Tabebuia* being misread as *Tabebuta* at the time that the plant was introduced to Myanmar, and the latter seems to have become the accepted Burmese name. The variant *d̪əbye*^2^ *bu*^2^ *ta*^1^ was recorded at one nursery and is based on the well-known *d̪əbye*^2^ (*Syzygium* spp.); this change may reflect an attempt to nativise an otherwise foreign-sounding name by merging it with a native Burmese label.

**Figure 1:**
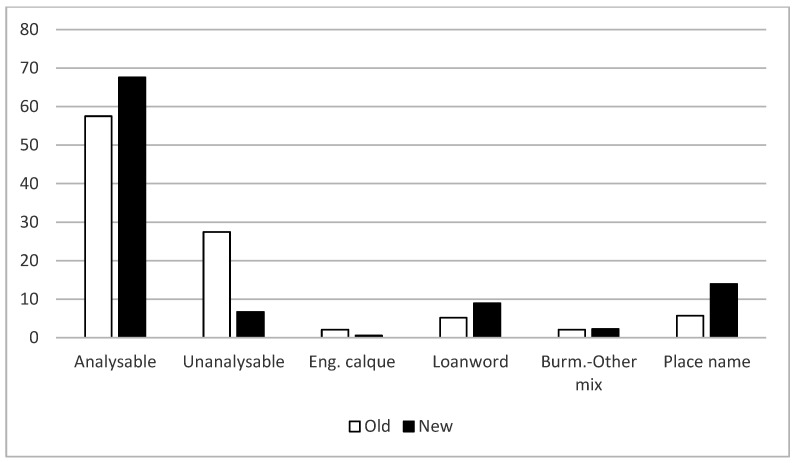
Proportions (%) of various naming strategies among older and newer ornamental plants. See text for details.

Quantitative analysis of the first six categories mentioned above shows that certain naming strategies are highly preferred over others (Figure 1). There are also statistically significant differences in the way older plants were named, in comparison to the more recent arrivals (χ^2^ = 32.8, d.f. = 3, *p* < 0.01). Note that Categories 3 and 5 were left out of the Chi Square analysis due to low count values (less than 5). Analysable (descriptive/allusive) names dominate among both older and newer plants, with 58% and 68% of names, respectively, following this strategy. While unanalysable names formed the second biggest category among the older plants (28%), the proportion of this name type was significantly reduced among the new plants (6%). Conversely, the proportion of plant names that include a place name was much higher among the newer arrivals (14%) than among the older plants.

Of the above categories, 1 to 5 are straightforward, and require no further explanation. Categories 6 and 7 are more interesting, as a number of ethnotaxa can be placed in both. As mentioned above, plants whose names indicate geographic origin are overrepresented among the newer arrivals. It is possible that some of the geographical indications have been included for marketing purposes, as the names suggest that the plants in question are from Korea, Thailand or Taiwan, whereas they are actually native to Myanmar. Examples include *yo*^3^ *dəya*^3^ *t̪iɁ min*^3^, *thain*^2^ *wan*^2^ *t̪iɁ min*^3^ ‘Thai-tree-king’, ‘Taiwan-tree-king’ for *Podocarpus macrophyllus* and *ko*^2^ *ri*^3^ *ya*^3^ *zun*^2^ ‘Korea-*zun*’ for *Wrightia religiosa*. According to POWO, both species are Myanmar natives, and it appears that epithets such as Korea and Taiwan have been used simply to make the plants seem more exotic, and to increase their appeal. In other cases, the geographical label does not seem to match the plant’s actual origins. One example is *jəpan*^2^ *bədauɁ* ‘Japan-*badauɁ*’ for *Galphimia gracilis*, which actually originates in South and Central America. This may once again be a marketing ploy, as Myanmar gardeners would be far more familiar with Japan than with Nicaragua or Peru. Alternatively, the name could reflect the country from which the plant was imported into Myanmar.

Several ethnotaxa that contain geographical labels are also lumped with one or more botanically unrelated taxa (Category 7 above). Further examples of this are presented in Table S3, along with ethnotaxa that do not contain geographic labels. The data clearly show that, of the 16 groups of ethnotaxa that share a ‘generic’ label and also contain at least one older and one newer ornamental plant, only 3 groups are botanically monogeneric (*Syzygium*, *Jasminum* and *Gardenia*). All other groups contain members from different genera and, in fact, from different botanical families. This means that the naming of new plants is mostly carried out on the basis of features other than biological relatedness to known plants.

A wide variety of orchids is currently available to hobby gardeners in Yangon, including showy cultivars developed overseas, as well as wild-harvested orchids from all over Myanmar. The naming of orchids is described in the following section.

### 2.2. Orchids

In the past, hobby gardeners used to cultivate a handful of orchid varieties: the most common include the spider orchid *Arachnis flos-aeris*, *A. maingayi*, the stick orchid *Cleisostoma simondii*, the yellow dancing lady orchid *?Cyrtochilum (Oncidium) flexuosum* x *sphacelatum* and a few varieties of *Cattleya labiata*. In addition, the iconic *d̪əzin*^2^ *ban*^3^ (*Bulbophyllum auricomum*), prized for its delicate beauty and fragrance, and worn by women in their hair, was also grown by orchid enthusiasts, along with a handful of native *Dendrobium* species (Table S4). The past two decades have seen a massive increase in the number of orchid varieties available to hobby gardeners, and many new Burmese names have been coined to accommodate these new ethnotaxa (Table S4). The vast majority of orchid names bear the head *t̪iɁ khwa*^1^ ‘orchid’, lit. ‘tree-straddle’. While it is possible to extend the name using *pin*^2^ ‘plant’ (and arrive at *t̪iɁ khwa*^1^ *bin*^2^ ‘orchid plant’), the morpheme *pin*^2^ is usually omitted in the names of individual orchid ethnotaxa.

Apart from *d̪əzin*^2^ *ban*^3^, practically all orchid names, both older and newer, are analysable and descriptive, alluding to the physical attributes of the orchid flower, or evoking some other positive association. Examples include the following:

*hsin*^2^*ma*^1^*myeɁ kwin*^3^*t̪iɁ khwa*^1^ ‘elephant-female-eye-orchid’ (*Dendrobium pulchellum* cf. *loddigesii*)

*daun*^3^*hmi*^3^*t̪iɁ khwa*^1^ ‘peacock-tail-orchid’ (*Dendrobium nobile*)

*pya*^3^*oun*^2^*t̪iɁ khwa*^1^ ‘bee-hive-orchid’ (*Gastrochilus calceolaris*)

In online orchid stores based in English-speaking countries, the common names for orchids are often translations of botanical names; this is particularly true of the more exotic, and possibly rarer, varieties, and it is not uncommon to see plants called Noble Dendrobium and Benson’s Vanda for sale. Burmese gardeners have been very proactive in coining imaginative and evocative names, at least for the ethnotaxa that occur locally. A similar phenomenon has been noted in neighbouring Thailand [13]; here too, most orchid names incorporate the morpheme *ueang* ‘orchid’ but at the beginning, and the names themselves are analysable and descriptive (Table 1). It is interesting to note a handful of convergences in the names given to some orchids; both Burmese and Thai growers have chosen to invoke elephant/buffalo eyes for *D. nobile* and a toothbrush for *D. secundum* (incidentally, the English name for this species is also *toothbrush orchid*). The names for *Rhynchostylis retusa* in Burmese and Thai translate as ‘cat tail’ and ‘squirrel tail’, respectively (*foxtail orchid* in English), whereas two unrelated orchids (*Gastrochilus calceolaris* and *Pholidota articulata*) are named for the resemblance of their inflorescences to bee/wasp nests in Burmese and Thai, respectively.

With a handful of exceptions, most of the orchid species listed in Table S4 are native to Myanmar, but this does not mean that introduced orchid species, varieties and cultivars are absent from the Myanmar horticultural scene. On the contrary, a large number of new orchid varieties, mostly imported from Thailand, can be purchased in physical and online stores in Yangon. These varieties, however, do not have any local Burmese names and, in physical stores, are sold in pots tagged with complex cultivar names (Genus, grex and cultivar names) of varying length. Customers are able to choose an orchid of their liking by consulting a colour printout showing thumbnail photographs of the orchid flowers, each labelled with a code known to the seller. In online shops as well, customers make their choices based on photographs of the orchid flowers, often without bothering to identify the type of orchid any more precisely than with broad generic labels, such as *dendro*, *vanda*, *dancing*, *leiɁ pya2* (*Phalaenopsis*, lit. ‘butterfly’) and so on.

## 3. Discussion

The last 20 years have seen a sharp rise in the number of new plants being sold in plant nurseries in Yangon. According to people working at plant nurseries, the main source of these plants is Thailand, where the development of new cultivars seems to be a major industry [14]. Many new species of native and non-native orchids are now commonly available, but among the non-orchid ornamentals, the proportion of exotic species has increased significantly. While many of the older plants are still being sold, some common species appear to have fallen out of favour and are now difficult to find. Yet others have been displaced by newer, more visually striking varieties and cultivars.

The now unpopular older plants have not disappeared completely from Yangon. Cultivars of *Acalypha hispida* and *A. wilkesiana*, *Coleus scutellaroides*, *Canna indica*, *Catharanthus roseus*, *Tagetes erecta* and *Zinnia elegans* can commonly be seen growing in public parks, outside official government buildings and in monastery compounds. However, finding a nursery that still sells these plants might be a challenge for the average hobby gardener, as they are now considered low-end ‘landscaping’ plants and are likely sold mostly by wholesale plant sellers located around the outskirts of Yangon. The first author (AS) of the present paper was once looking to buy a *P. lutea* to grow at home but was told by staff at one of the nurseries visited during the course of this study that the plant was no longer sold there, because nobody wanted it. On another occasion, AS saw a solitary variegated *C. scutellaroides* at a nursery while buying some other plants and asked the shopkeeper how much it cost. The latter responded that AS could have it for free; again, presumably, because nobody wanted it. In contrast to public parks and monastery gardens, high-end hotels and other large private institutions in Yangon tend to favour newer, more upmarket plants for their landscaping, such as large bamboos and palms for architectural elements and *Aglaonema* spp., *Philodendron* spp. and *Equisetum hyemale* among the smaller species. Sometimes, private garden fashions and hotel garden fashions seem to go hand-in-hand. The use of a particular thin-stemmed bamboo by the once-Singapore-owned Sedona hotel (the first large international hotel to open in Yangon in 1996) became so well known that that bamboo variety came to be known as *si*^2^ *do*^3^ *na*^3^ *wa*^3^ ‘Sedona bamboo’ and was highly sought after for a time.

Nursery owners in Myanmar have kept up with the influx of new plants by enthusiastically coining new names in clever and imaginative ways. Many of the strategies noted by [15] for the Indigenous languages of northwestern North America also apply to Burmese. These include outright borrowings, reference expansion of an existing plant (based on perceived similarities with a new plant) and the coining of a completely new name based on the new plant’s notable features. In addition, the vast majority of Burmese plant names, both older and newer, are analysable, highly descriptive or evocative names. Many of the newer leafy ornamentals have names with positive connotations or the suggestion that the plants in question are symbols of auspiciousness or bringers of luck and prosperity. Examples include *ein*^2^ *dɔ*^2^ *min*^2^ *gəla*^2^ *gəmoun*^3^ (house-royal-prosperity-*gəmoun*) ‘*Aglaonema* sp.’, *thi*^2^ *pauɁ pin*^2^ (lottery-win-plant) ‘*Pachira aquatica*’ and *shwe*^2^ *hmoun*^2^ *ngwe*^2^ *hmoun*^2^ *gəmoun*^3^ (gold-dust-silver-dust-*gəmoun*) ‘*Philodendron* ‘Birkin’’. In contrast, the proportion of unanalysable names is far lower among the newer plants. It is likely that the proportion of unanalysable names (such as *gan*^2^ *dəma*^2^ ‘*Chrysanthemum* sp.’ and *moun*^2^ *dain*^2^ ‘*Cycas* sp.’) is higher among the older plants, because they represent either old indigenous compound words or words that were naturalised long ago. In both cases, the historic compositional meaning may have been lost over time [16], resulting in an unanalysable (to present-day Burmese speakers) name. Naturally, this explanation does not apply to monosyllabic names such as *ngu*^1^ ‘*Cassia fistula*’ and *wa*^3^ ‘bamboo’, which are only found among the older plants, but it does provide a compelling reason for why longer unanalysable names are rare among the newer plants. The newer plant names were only coined recently, and any compositionality inherent in the names is still accessible to Burmese speakers.

The number and variety of orchids available in Yangon has increased significantly in recent years, with many native and exotic species and cultivars entering the market. While several unique and evocative Burmese names have been coined for the native orchids, the exotics tend to be referred to by broad labels of convenience, such as *catteleya*, *dendro* and *vanda.* This is not a new practice, however, as indicated by the use of the category label *vanda*, along with the more precise Burmese name *mo*^3^ *loun*^3^ *hmain*^3^ (sky-all-hazy) for the spectacular blue native *Vanda coerulea* in a popular song from 1985:

မိုးလုံးမှိုင်းလို့တို့သိခဲ့တယ်ဗင်ဒါတွေ

ကောင်းကင်ယံထက်ကျွန်းထိပ်ဖျားကိုတွယ်ကာနေ

နွေအခါဆိုရင်ရွက်ရိုးဆစ်မှာဖူးငုံလှိုင်ပွင့်စေ။

Ko Mun Aung (1985)

The *vandas* that we used to call *mo*^3^ *loun*^3^ *hmain*^3^

Reach to the sky from the tops of teak trees,

In the summer, may buds bloom aplenty from between their leaves.

### 3.1. Ornamental Names and Ethnobiological ‘Universals’

There is evidence, among both the older and newer ethnotaxon names, of violations of some of Berlin’s [11] nomenclatural and classificatory ‘universals’. In particular, Burmese ethnotaxa from both categories frequently do not align with scientific genera. There are, of course, instances where Berlin’s prediction seems to hold: bamboos are all called *wa*^3^ (along with a specific-level epithet, such as *shwe*^2^ *wa*^3^ ‘golden bamboo’), and similarly, the two *Bauhinia* spp., two *Clematis* spp., two *Crinum* spp., three *Jasminum* spp. (but not *J. auriculatum* and *J. grandiflorum*), two out of three *Gardenia* spp., three *Plumeria* spp. and two out of four *Dracaena* spp. share the same Burmese generic ethnotaxon label (Table S1). However, there are at least as many instances of the contrary listed in Table S1; these include four *Euphorbia* spp., two *Gardenia* spp., two *Globba* spp., two *Hedychium* spp., two *Heliconia* spp., two *Lagerstroemia* spp., two *Magnolia* spp., two *Terminalia* spp. and two *Wrightia* spp.

Conversely, there are numerous polytypic Burmese ethnotaxa which contain plants from two or more genera; the group of plants that includes *zəgəwa*^2^ (*Magnolia champaca*, yellow), *zəgəphyu*^2^ (*M. champaca*, white), *zəgəni*^2^ (*M. figo*), *zəgəzein*^3^ (*Cananga odorata*), *male*^3^ *zəga*^3^ (*Adenium obesum*) and *tayouɁ zəga*^3^ (*Plumeria alba, P. rubra*, *P. obtusa*) all contain the generic label *zəga*^3^. While the genus *Magnolia* dominates the group with three ethnotaxa, there are other *Magnolia* species (namely *M. compressa*, *M. liliiflora*, *M. globosa* and *M. kobus*) which have completely unrelated Burmese names that do not contain *zəga*^3^. Therefore, it is not possible to map the ethnotaxon *zəga*^3^ onto a single biological genus or the genus *Magnolia* onto a single ethnotaxon. Note that this set of names shows certain peculiarities that can only be explained by the rules of Burmese morphosyntax and morphophonology, and not by Berlin’s model. First, the head *zəga*^3^ of the plant names can occur word-initially or word-finally, as has been noted in previous studies [17]. Second, the pronunciation of the *zəga*^3^ element also changes (the second syllable changes from tone three to a toneless schwa) when it appears word-initially. Third, and most importantly, there is no plant labelled only by the generic *zəga*^3^, contrary to Berlin’s categorisation principles at the generic–specific interface (i.e., if there is an *English oak* and a *sessile oak*, there should also be an *oak*). Finally, as intimated in the Notes to Table S1, the above plant names are actually incomplete, as most Burmese plant names (as well as most fish names and some bird names) obligatorily take a superordinate label (*plant, fish and bird*, respectively) as the head morpheme of the name [18]. Thus, strictly speaking, a yellow-flower-bearing *Magnolia champaca* tree would be called *zəgawa*^2^ *bin*^2^ ‘*zəgawa*^2^ tree’. This is a violation of the ‘universal’ that folk generic names be primary names.

Similar levels of mismatch are seen in the naming of the popular leafy ornamentals *pein*^3^ and *gəmoun*^3^. *Pein*^3^ appears most commonly in the names of species of *Alocasia*, *Colocasia* and *Anthurium*, but the name can also be extended to include *Caladium bicolor.* Interestingly, Burmese speakers distinguish between *Alocasia/Colocasia* on the one hand (grown for their foliage) and *Anthurium* on the other (grown for its flowers) by adding the head morphemes *pin*^2^ ‘plant’ and *pan*^3^ ‘flower’ to the name. Thus, *pein*^3^ *bin*^2^ usually indicates *Alocasia/Colocasia*, while *pein*^3^ *ban*^3^ is the normal label for *Anthurium*. The case of the numerous foliage plants called *gəmoun*^3^ is far more extreme. Tables S1 and S2 contain around 50 ethnotaxa whose names include the name *gəmoun*^3^, with most being used to label members of the genera *Aglaonema*, *Dieffenbachia*, *Spathiphyllum* and *Philodendron* (Araceae) and *Goeppertia* (Marantaceae). Counting all the instances of *gəmoun*^3^, we find that the name is used to label 18 genera across 7 families.

### 3.2. Future Directions

Data from Myanmar are often underrepresented in, or completely missing from, global biodiversity databases, possibly due to the decades of political strife that have caused the country to be isolated from the outside world. The POWO is no exception, and many plants that are commonplace in Myanmar, or even those that are listed in [19], are indicated as ‘not present’ in the database. The fact that the majority of the orchids mentioned in Table S4 are listed as ‘native’ in POWO is probably a reflection of the greater levels of research effort expended on Orchidaceae in general, in Myanmar and elsewhere. In any case, the data presented above show a clear increase in the numbers of exotic species being sold in Yangon nurseries. This has potentially serious ecological implications, as escaped horticultural species can be responsible for biological invasions that reduce local biodiversity [20,21]. Most studies on biological invasions have been carried out in temperate parts of the world, but it is possible that tropical regions may be nearly as susceptible to invasions, especially in disturbed ecosystems [22]. It is, therefore, a matter of some urgency to first identify any escaped exotics in Myanmar and to investigate the impacts of such escapes on local ecosystems.

The recent boom in the trade of native orchids is also a cause for concern, as not only are most such plants wild collected, but many are also likely to be protected species [13]. Moreover, wild-collected orchids from Myanmar make up a significant proportion of the orchids sold at markets in neighbouring Thailand, with an estimated 15% of Myanmar’s known orchid flora being represented among the species sold [23]. Worryingly, orchid harvesters from Myanmar frequently reported that they collected all species indiscriminately, and that they had noticed declines in the abundances of all species [13,23]. As a first step, online traders, nursery owners and orchid enthusiasts in Myanmar need to be educated about the negative impacts of collecting native orchids in the wild. Subsequent measures, which are contingent on the return of peace and stability to the country, should include increased legislation for the protection of threatened species of all kinds, as well as effective enforcement of that legislation. Research efforts towards the propagation of rarer and/or more commonly traded orchid species would also help to safeguard orchid biodiversity in Myanmar.

## 4. Materials and Methods

Plant names in Burmese were documented through a series of open-ended interviews with gardeners and nursery owners. Informed consent was obtained orally prior to data collection. Information on plants that were commonly grown in the 1980s and 1990s was obtained from five enthusiastic hobby gardeners (all female and all aged above 60 years), drawn from the authors’ circle of acquaintances. All five respondents lived in free-standing houses set within gardens (front and/or back) with an area of 40 m^2^–100 m^2^. Data collection involved free-listing and open-ended as well as semi-structured interviews; these are methods frequently used in ethnobotanical investigations [24,25]. The free-listing task and open-ended interviews were carried out at the respondents’ homes, where they were asked to list the plants that were grown in their gardens (by their parents or by themselves) when they were younger. At this stage, respondents were also encouraged to share their recollections of past gardening practices in general. Next, data collection continued in the respondents’ present-day gardens, where they were asked to name each plant currently growing there and to comment on its status, in terms of whether it was an ‘older’ or ‘newer’ plant. If a plant was labelled as ‘newer’, consultants were asked to estimate when it appeared on the horticultural market in Yangon and where it might have originated from. The names of plants that are currently in vogue were recorded by interviewing employees at three major plant nurseries located in different parts of Yangon (Bahan Township, Yankin Township, North Dagon Myothit Township). The owner of a fourth nursery (South Dagon Myothit Township) that primarily sold wild-collected orchids was also interviewed. Finally, supplementary information relating to the latter category was obtained from the Facebook accounts of four online plant shops, two of which specialised in orchids.

The plant names recorded in the interviews were transcribed and translated at a later date. The published resources [19,26] were used to locate any missing Burmese names and scientific identifications, while the online databases Plants of the World Online (POWO, URL: https://powo.science.kew.org/ (accessed on 5 December 2024)), Flora and Fauna Web (for Singaporean species, URL: https://www.nparks.gov.sg/florafaunaweb (accessed on 12 December 2024)) and Flowers of India (https://www.flowersofindia.net/ (accessed on 12 December 2024)) provided location information and English common names.

Statistical analyses were carried out using the online statistical platform Social Science Statistics (http://www.socscistatistics.com (accessed on 20 December 2024)).

## 5. Conclusions

The results of this study have revealed a significant shift in Yangon gardeners’ taste in ornamental plants over the last few decades. Many new variegated leafy ornamentals, as well as native and imported orchids, are now widely available, while some of the older plants that were once ubiquitous in people’s homes are now found mostly in very specific locations, such as monastery gardens, government offices, urban parks and low-end shopping venues. Plant sellers and gardeners have developed a large number of new, descriptive Burmese names to label the newcomers, resulting in mismatches in categorisation between Burmese ethnotaxa and biological taxa. This phenomenon is probably widespread among the world’s languages and should be investigated further. Finally, plant enthusiasts need to be made aware of key ecological issues such as the escape of exotic plants, with the potential to become weeds, and the biodiversity loss associated with the wild harvest of native orchid species.

## Figures and Tables

**Table 1:** A selection of wild-collected Thai orchids and their names from [13]. Name translations by Toop Rodsuti, Canberra. Note that the Thai *ueang* means ‘orchid’.

Latin Name	Latin Transcription of Thai Name	Translation of Thai Name
*Vanda (Ascocentrum) ampullacea* (Roxb.) L.M.Gardiner	khem muang	purple needle
*Vanda (Ascocentrum) curvifolia* (Lindl.) L.M.Gardiner	khem daeng	red needle
*Vanda (Ascocentrum) garayi* (Christenson) L.M.Gardiner	khem saet	orange needle
*Bulbophyllum blepharistes* Rchb.f.	singto samoa hin	anchor stone lion
*Cleisostoma arietinum* (Rchb.f.) Garay	khao phae	goat horns
*Dendrobium chrysotoxum* Lindl.	ueang kham	orchid female.name
*Dendrobium falconeri* Hook.	ueang sai wisut	curtain line orchid
*Dendrobium findlayanum* C.S.P.Parish & Rchb.f.	ueang phuang yok, wai pom	jade orchid, rattan knot
*Dendrobium jenkinsii* Wall. ex Lindl.	ueang phung noi	little bee orchid
*Dendrobium lindleyi* Steud.	ueang phung	bee orchid
*Dendrobium pachyphyllum* (Kuntze) Bakh.f.	ueang song bai	two-leaf orchid
*Dendrobium parishii* H. Low	ueang sai nam khrang	shellac river orchid
*Dendrobium pulchellum* Roxb. ex Lindl.	ueang chang nao, ueang kham ta khwai	elephant orchid, buffalo eyes orchid
*Dendrobium secundum* (Blume) Lindl. ex Wall.	ueang praeng sifan	toothbrush orchid
*Dendrobium senile* C.S.P.Parish & Rchb.f.	ueang chani	gibbon orchid
*Dendrobium sulcatum* Lindl.	ueang champa nan	Nan Province champakaflower orchid
*Grammatophyllum speciosum* Blume	wan phetchahung	?storm orchid/plant
*Habenaria carnea* Gower	ueang lin mankorn	dragon’s tongue orchid
*Phalaenopsis cornu-cervi* (Breda) Blume & Rchb.f.	khao kwang on	young deer antlers
*Phalaenopsis pulcherrima* (Lindl.) J.J.Sm.	ma wing, daeng ubon, ma bin	running horse, red lotus, flying horse
*Coelogyne (Pholidota) articulata* (Lindl.) Rchb.f.	ueang rangtaw	wasp nest orchid
*Rhynchostylis gigantea* (Lindl.) Ridl.	chang kra, chang daeng (red), chang phueak (white)	elephant freckles, red elephant, white elephant
*Rhynchostylis retusa* Blume	(hang kraw rok)	squirrel tail

## Data Availability

The original contributions presented in this study are included in the article. Further inquiries can be directed to the corresponding author(s).

## Supplementary Materials

The following supplementary information can be downloaded at: https://www.mdpi.com/article/10.3390/plants14040552/s1; Table S1: Ornamental plants that were commonly grown in private gardens in the 1980s and 1990s; Table S2: Newer ornamental plants that are currently being sold in physical or online stores in Yangon, Myanmar; Table S3: New plant names based on pre-existing ones; Table S4: Older and newer orchid varieties grown in Yangon, Myanmar. References [27,28,29] are cited in the supplementary materials.
