# Supplementary material for: Changing Perceptions of Ornamental Plants in Urban Yangon, Myanmar
Source: Plants (Basel). 2025 Feb 11;14(4):552. doi: 10.3390/plants14040552 (PMC11859482; doi:10.3390/plants14040552)
Supplement: Supplementary file 1 [file plants-14-00552-s001.zip › plants-3453632-supplementary.pdf]

Table S1. Ornamental plants that were commonly grown in private gardens in the 1980s and 1990s; species that are still sold in plant nurseries are marked with *.

Family	Latin name[1]	Pr.[2]	Local name[3]	Transcription[4]	Translation of local name	English name
Acanthaceae	*Crossandra infundibuliformis* (L.) Nees	i	မိုးဆွေပန်း	mo^{3} hswe^{2} ban^{3}		firecracker flower
	Pachystachys lutea Nees	i	နဂါးမောက်ရွှေ	nəgəmauʔ shwe^{2}	dragon-crest-gold	yellow shrimp plant
	Pseuderanthemum carruthersii (maculatum) (G.Lodd.) I.M.Turner	i	?			Carruthers' falseface
	Pseuderanthemum crenulatum (Wall. ex Lindl.) Radlk.	y	?			none
	**Ruellia simplex* C. Wright	i	စင်ဒရဲလားပန်း	sin^{2} dərɛ^{3} la^{3} ban^{3}	Cinderella-flower	desert petunia
	**Thunbergia grandiflora* Roxb., **T. laurifolia* Lindl.	y	နွယ်ညို	nwɛ^{2} nyo^{2}	vine-brown (also the name for *Wisteria*)	blue trumpet vine, Bengal clock vine
Alismataceae	**Aquarius (Echinodorus) cordifolius* (L.) Christenh. & Byng	n	ရေစံပယ်	ye^{2} zəbɛ^{2}	water-jasmine	creeping burhead
Amaranthaceae	*Alternanthera brasiliana* (L.) Kuntze (purple)	n	ပုလဲသွယ်	pəlɛ^{3} t̪wɛ^{2}		Joseph's coat
	Celosia argentea L.	i	ကြက်မောက်ပန်း(အဖြူ)	cɛʔ mauʔ pan^{3} (əphyu^{2})	cock-comb-flower (white)	cockscomb
Amaryllidaceae	*Crinum asiaticum* L.	y	ကိုယ်ရံကြီး	ko^{2} yan^{2} ji^{3}		spider lily
	Crinum x *amabile*		ကိုယ်ရံကြီးအနီ	ko^{2} yan^{2} ji^{3} (əni2)		spider lily
	**Hippeastrum puniceum* (Lam.) Voss	n	လေးကျွန်းကြာခိုင်, လေးကျွန်းစကြာ	le^{3} cun^{3} ca^{2} khweʔ, le^{3} cun^{3} seʔ ca^{2}		Barbados lily

	Hymenocallis littoralis (Jacq.) Salisb., *H. speciosa* (L.f. ex Salisb.) Salisb.	n	?			beach spider lily
	**Scadoxus multiflorus* (Martyn) Raf.	n	ဘောလုံးပန်း	bɔ^{3} loun^{3} ban^{3}	ball-flower	ball lily
	**Zephyranthes carinata* Herb.	n	နှင်းပန်း	hnin^{3} ban^{3}	snow-flower	pink zephyr lily
	**Zephyranthes citrina* Baker	i	နှင်းပန်း	hnin^{3} ban^{3}	snow-flower	yellow rain lily
Annonaceae	**Cananga odorata* (Lam.) Hook.f. & Thomson	y	စံကားစိမ်း	zəgəzein^{3}	*zəga*-green	ylang ylang
	**Desmos chinensis* Lour. (red)	y	စကားစိမ်းပန်းအနီရောင်	zəgəzein^{3} ban^{3} əni^{2} yaun^{2}	*zəga*-green-red-colour	dwarf ylang ylang
	**Monoon (Polyalthia) longifolium* (Sonn.) B.Xue & R.M.K.Saunders	i	ရေတမာ, သင်္ဘောတည်	ye^{2} təma^{2}, t̪in^{3} bɔ^{3} dɛ^{2}	water-tamarind	(false) ashok, mast tree
Apocyanaceae	**Adenium obesum* (Forssk.) Roem. & Schult.	n	မလေးရှားစကား, ကန္တာရနှင်းဆီ	məle^{3} sha^{3} zəga^{3}, gan^{2} da^{2} ya^{1} hnin^{3} zi^{2}	Malaysia-*zəga*, desert-rose	desert rose
	**Allamanda blanchetii* A.DC	n	ခရာညို	khəya^{2} nyo^{2}	*khəya*-brown	purple allamanda
	Cascabela thevetia (L.) Lippold *(Thevetia peruviana)*	n	၁၂ရာသီပန်း	12 ya^{2} d̪i^{2} pan^{3}	12-month-flower	yellow oleander
	Catharanthus roseus (L.) G.Don (pink, white)	i	သင်္ဘောမညိုး, ခရာနီ	t̪in^{3} bɔ^{3} məhnyo^{3}, khəya^{2} ni^{2}		Madagascar periwinkle
	**Nerium oleander* L.	y	နွယ်သာကီ	nwɛ^{2} t̪a^{2} ki^{2}		oleander
	**Plumeria alba* L.	n	တရုတ်စကား	təyouʔ zəga^{3}	China-*zəga*	frangipani
	**Plumeria obtusa* L.	n	တရုတ်စကား	təyouʔ zəga^{3}	China-*zəga*	frangipani
	Plumeria pudica Jacq.	n	?			wild plumeria
	**Plumeria rubra* L.	n	တရုတ်စကား	təyouʔ zəga^{3}	China-*zəga*	frangipani, red pagoda tree
	**Tabernaemontana divaricata* (L.) R.Br. ex Roem. & Schult.	y	ဇလပ်စကြ်ာ	zəlaʔ seʔ ca^{2}		pinwheel flower, crepe jasmine

	Vallaris solanacea (Roth ex Roem. & Schult.) Kuntze	y	ခင်ဘုတ်	khin^2 bouʔ		bread flower
Araceae	*Aglaonema commutatum* Schott	n	စီးပွားဂမုန်း	si^3 bwa^3 ye^3 gəmoun^3	trade-*gəmoun*	poison dart plant
	Aglaonema nitidum (Jack) Kunth (green variegated)	y	ဂမုန်း	gəmoun^3		Burmese evergreen
	**Alocasia indica* (Lour.) Spach	y	ပိန်းပင်	pein^3 bin^2		Indian taro
	**Alocasia macrorrhizos* (L.) G.Don	n	ဆင်နရွက်ပိန်း	hsin^3 nəywɛʔ pein^3	elephant-ear-*pein*	giant taro, elephant ear taro
	**Alocasia sanderiana* W.Bull	n	အင်းဂမုန်း	in^3 gəmoun^3	lake-*gəmoun*	kris plant
	**Anthurium andraeanum* Linden ex André (red, not variegated, small and large forms)	n	ပိန်းပန်း	pein^3 ban^3	*pein*-flower	painter’s palette
	Caladium bicolor (Aiton) Vent. (green, purple, white spots)	n	ပါတိတ်ပိန်း	pa^2 teiʔ pein^3	batik-*pein*	angel wings, heart of Jesus
	**Dieffenbachia* sp. (large, green, unvariegated)		မယ်တော်ကြီးဂမုန်း	mɛ^2 dɔ^2 ji^3 gəmoun^3	female.saint-*gəmoun*	dumb cane
	**Dieffenbachia seguine (amoena)* (Jacq.) Schott (green variegated; white with green border)	n	ဘိုးတော်ဖြူ	bo^3 dɔ^2 phyu^2 gəmoun^3	male.saint-white-*gəmoun*	dumb cane
	**Dieffenbachia* sp. (mottled white lime-green, white stem)		ကျိကျိတက်ဂမုန်း	ci^1 ci^1 teʔ gəmoun^3	extremely.wealthy-*gəmoun*	dumb cane
	**Epipremnum aureum* (Linden & André) G.S.Bunting	n	ရွှေလာငွေလာ	shwe^2 la^2 ngwe^2 la^2	gold-come-silver-come	money plant, pothos
	**Monstera adansonii* Schott	n	ရွက်ပေါက်ဂမုန်း	yweʔ pauʔ gəmoun^3	leaf-perforated-*gəmoun*	Swiss cheese plant, monkey mask plant
	**Monstera deliciosa* Liebm.	n	ကျားလက်ဝါး	ca^3 leʔ wa^3	tiger-palm	Swiss cheese plant
	**Philodendron* sp.		?			philodendron
	*?*Spathiphyllum* ‘sensation’	n	ရှင်သီဝလိဂမုန်း	shin^2 t̪i^2 wəli^1 gəmoun^3	monk.name-*gəmoun*	peace lily

	**Spathiphyllum wallisii* Regel	n	ရှင်သဝလိဂမုန်းအသေးစား	shin^{2} t̪i^{2} wəli^{1} gəmoun^{3} ət̪e^{3} za^{3}	monk.name-*gəmoun*-small	peace lily
	**Syngonium podophyllum* Schott	n	စင်ကူး(နီးယား)	E: sin^{2} ku^{3} (ni^{3} ya^{3})		arrowhead plant
Araliaceae	**Polyscias fruticosa* Harms	i	ကြောင်လက်သဲ, ဂဠုန်လက်သဲ	caun^{2} leʔ t̪ɛ^{3}, gəloun^{2} leʔ t̪ɛ^{3}	cat-claw, garuda-claw	Ming aralia
Araucariaceae	*Araucaria heterophylla* (Salisb.) Franco, *A. bidwillii* Hook., *A. cunninghamii* Mudie, **A. columnaris* (G.Forst.) Hook.	n	ထင်းရှူးပဒေသာ	thin^{3} yu^{3} bəde^{2} t̪a^{2}	pine-assortment	Norfolk island pine
Arecaceae	**Chamaedorea elegans* Mart.	n	lucky bamboo			parlour palm
	Livistona chinensis (Jacq.) R.Br. ex Mart., **Livistona jenkinsiana* Griff.	n, y	တောထန်း	tɔ^{3} than^{3}	forest-toddy.palm	Chinese fan palm
	**Rhapis excelsa* (Thunb.) A.Henry	n	ကြိမ်စပ်	cein^{2} saʔ	cane-hybrid	lady palm
	**Chrysalidocarpus (Dypsis) lutescens* H.Wendl.	n	ယိုးဒယား အုန်းပွား	yo^{3} dəya^{3} oun^{3} bwa^{3}	Thai-coconut-spreading	golden cane palm
Asparagaceae	**Chlorophytum comosum* (Thunb.) Jacques (middle stripe white)	n	မျက်ကြား	myeʔ ca^{3}	grass-striped	spider plant
	**Cordyline fruticosa* (L.) A.Chev. (green)	i	ဇော်ဂျီတောင်ဝှေး	zɔ^{2} ji^{2} taun^{2} hwe^{3}	mythical.being-magic.staff	ti plant
	**Dracaena (Sansevieria) trifasciata* (Prain) Mabb. (tall, dwarf)	i	နဂါးစက်, နဂါးစပ်	nəgəzeʔ, nəgəzaʔ		mother-in-law’s tongue
	**Dracaena fragrans* (L.) Ker Gawl.	n	ဇော်ဂျီတောင်ဝှေး, လပ်ကီးတိုင်, တရုတ်မန်းနီးပင်, ဒန့်ဒကူး, lucky bamboo	zɔ^{2} ji^{2} taun^{2} hwe^{3}, laʔ ki^{3} tain^{2}, təyouʔ man^{3} ni^{3} pin^{2}, dan^{1} dəku^{3}	mythical.being-magic.staff, lucky-pole, China-money-plant	corn(stalk) plant
	**Yucca* sp.	n	သစ်မင်း	t̪iʔ min^{3}	tree-king	yucca
Asphodelaceae	**Aloe vera* (L.) Burm.f.	n	ရှားစောင်းလက်ပတ်	sha^{3} zaun^{3} leʔ paʔ		aloe vera

	Hemerocallis sp.	n	?			day lily
Aspleniaceae	**Asplenium nidus* L.	n	ငှက်သိုက်	hngeʔ t̪aiʔ	bird-nest	bird's nest fern
Asteraceae	*Chrysanthemum indicum* L., **Chrysanthemum x morifolium*	n	ဂန္ဓမာ	gan^2 dəma^2		chrysanthemum
	**Cosmos sulphureus* Cav.	i	?			cosmos
	**Helianthus annuus* L.	n	နေကြာပန်း	ne^2 ja^2 ban^3	sun-lotus-flower	sunflower
	Sphagneticola trilobata (L.) Pruski *(Wedelia trilobata)*	y	ပန်းဗိဇပ်	pan^3 bi^1 zaʔ	flower-*Chromolaena*	creeping ox-eye
	Tagetes erecta L.	i	ထပ်တရာပန်း, ဒေဝါလီပန်း	thaʔ təya^2 pan^3	layer-hundred-flower	marigold
	Zinnia elegans Jacq.	i	ဒေါင်းဖန်ဝါ	daun^3 phan^2 wa^2		zinnia
Balsaminaceae	**Impatiens balsamina* L.	i	ဒန်းပန်း	dan^3 təleʔ pin^2		rose balsam
Begoniaceae	**Begonia* sp.		ကြွေပန်း	cwe^2 ban^3	porcelain-flower	begonia
	Campsis (Tecoma) radicans (L.) Bureau	n	?			trumpet vine
	**Dolichandra unguis-cati* (L.) L.G.Lohmann	n	မင်္ဂလာနွယ်, ?နွယ်နှောင်း	min^2 gəla^2 nwɛ^2, nwɛ^2 hnaun^3	auspicious-vine	cat's claw creeper
	**Jacaranda mimosifolia* D.Don	n	စိန်ပန်းပြာ	sein^2 ban^3 bya^2	diamond-flower-blue	jacaranda
	**Pyrostegia venusta* (Ker Gawl.) Miers	n	သော်ကတံတိုင်း	t̪ɔ^2 ka^1 dədain^3		flame vine, flaming trumpet
	**Radermachera sinica* (Hance) Hemsl.	y	စိတ်ရွှင်လန်း, ပိတောက်နီ	seiʔ shwin^2 lan^3, bədauʔ ni^2	spirit-joyful, *bədauʔ*-red	China doll
	**Tabebuia aurea* (Silva Manso) Benth. & Hook.f. ex S.Moore	n	တပေဘူတ, သပြေဘူတ	təbe^2 bu^2 ta^1, d̪əbye^2 bu^2 ta^1		yellow trumpet tree
	**Tecoma stans* (L.) Juss. ex Kunth	i	စိန်တချူ	sein^2 dəju^2	fruit.picker	yellow bells
Bignoniaceae	*Millingtonia hortensis* L.f.	n	ဧကရာဇ်	e^2 kəriʔ	emperor	tree jasmine, Indian cork tree

Bromeliaceae	*Bromeliaceae		ပန်းနာနတ်	pan^{3} na^{2} naʔ	flower-pineapple	bromeliads
Cactaceae	**Epiphyllum oxypetalum* (DC.) Haw.	n	ကုမုဒြာ	ku^{1} mu^{1} dra^{2}		orchid cactus, queen of the night
	?Schlumbergera truncata (Haw.) Moran *L.*	n	?			Christmas cactus
Calophyllaceae	**Calophyllum inophyllum*	y	ပုန်းညက်	poun^{3} nyɛʔ		tamanu tree, Alexandrian laurel
	**Mesua ferrea* L.	y	ကံ့ကော်	gan^{1} gɔ^{2}		Ceylon ironwood
Cannaceae	*Canna indica* L.	i	ဗုဒ္ဓံသရဏံ	P: bouʔ dan^{2} t̪ərənan^{2}	Buddha-refuge (Pali prayer)	Indian shot
Caryophyllaceae	**Dianthus caryophyllus* L.	n	ဇော်မွှားပန်း	zaw^{2} hmwa^{3} ban^{3}		carnation
Celastraceae	*?*Siphonodon celastrineus* Griff.	y	ပန်းလိမ္မော်အကြား	pan^{3} lein^{2} mɔ^{2} əca^{3}	flower-lemon-striped	ivorywood
Chloranthaceae	**Chloranthus serratus* (Thunb.) Roem. & Schult.	n	၁ယူဇနာမွှေး	1 yu^{2} zəna hmwe^{3}	one-yojana.away-fragrant	chloranthus
Combretaceae	**Combretum indicum* (L.) DeFilipps	y	ထားဝယ်မှိုင်း	dəwɛ^{2} hmain^{3}		Rangoon creeper
Commelinaceae	*Cyanotis ciliata* (Blume) Bakh.f.	y	?			none
	Tradescantia spathacea Sw.	n	မီးကွင်းဂမုန်း	mi^{3} gwin^{3} gəmoun^{3}		oyster plant
Convolvulaceae	**Argyreia nervosa* (Burm.f.) Bojer	y	ကန်စွန်းကြီး	kəzun^{3} ji^{3}	kankong-big	elephant creeper
	**Ipomoea quamoclit* L.	i	မြတ်လေးနီ	myaʔ le^{3} ni^{2}		cypress vine
	**Poranopsis paniculata* (Roxb.) Roberty	y	ဆောင်တော်ကူး	hsaun^{2} dɔ^{2} ku^{3}		bridal bouquet
Costaceae	**Hellenia speciosa* (J.Koenig) S.R.Dutta	y	ဖလံတောင်ဝှေး	phəlan^{2} taun^{2} hwe^{3}		crepe ginger
Crassulaceae	*Crassula ovata* (Mill.) Druce	n	?			jade plant
	Echeveria gibbiflora DC	n	?			echeveria
	Kalanchoe crenata (Andrews) Haw.	n	ရွက်ကျပင်ပေါက်	yweʔ ca^{1} pin^{2} bauʔ	leaf-fall-plant-sprout	cathedral bells, kalanchoe

	Kalanchoe pinnata (Lam.) Pers.	i	ရွက်ကျပင်ပေါက်	ywe? ca¹ pin² bau?	leaf-fall-plant-sprout	cathedral bells, kalanchoe
Cupressaceae	**Platycladus (Thuja) orientalis* (L.) Franco	n	ရွှေဝါကျောက်ခက်	shwe² wa² cau? khe?		thuja, oriental arborvitae
Cycadaceae	**Cycas* sp. *(?circinalis, ?rumphii)*	n	မုန်တိုင်	moun² dain²		cycads
Cyperaceae	*Cyperus scariosus* R.Br	n	နွားမြက်ရင်း	nwa³ mye? yin³		umbrella grass
Dilleniaceae	**Dillenia indica* L.	y	အင်ကြင်းဖြူ, သဗြူသီး	in² jin³ phyu², d̪əbyu² d̪i³	*injin*-white	elephant apple
Dioscoreaceae	**Tacca chantrieri* André	y	ဇော်ဂျီမုတ်ဆိတ်	zɔ² ji² mou? hsei?	mythical.being-beard	black bat flower
Elaeocarpaceae	*?Elaeocarpus floribundus* Blume	y	သစ်ဖွဲ	t̪i? phwɛ³		Indian olive
Euphorbiaceae	*Acalypha hispida* Burm.f.	n	ကြောင်မြီး	caun² hmi³	cat-tail	cat’s tail
	Acalypha wilkesiana Müll.Arg.	n				copperleaf plant
	**Codiaeum variegatum* (L.) Rumph. ex A.Juss. (red-green-yellow veins, ‘gold dust’, thin-leaved)	n	ရွက်လှ, မင်းသားကြီး	ywe? hla¹, min³ d̪a³ ji³	leaf-beautiful, prince-great	croton
	Euphorbia milii Des Moul.	n	ကစ်မီ (kiss me quick), ရှားစောင်းသင်္ချိုင်း	kɪs mi³, sha³ zaun³ t̪in² gənei?	kiss.me,	kiss me quick (cf. *Portulaca pilosa*)
	Euphorbia pulcherrima Willd. ex Klotzsch	n	ခရစ္စမတ်ပန်း, ဂနိုင်ကျက်သရေ	khəri? səma? pan³, gənain¹ ce? t̪əye²	Christmas-flower, forest-charm	poinsettia
	Euphorbia tirucalli L.	i	ရှားစောင်းလက်ညှိုး, အရိုးဆေးပင်	sha³ zaun³ le? hnyo³, əyo³ hse³ bin²	aloe-finger, bone-medicine-plant	pencil cactus
	**Euphorbia (Pedilanthus) tithymaloides* L.	n	ဂုံဂမန်	goun² gəman²		slipper plant, devil’s backbone
	**Jatropha podagrica* Hook.	n	တပင်ရွှေထီး	dəbin² shwe² thi³	king’s.name	Buddha belly plant

Fabaceae	*Acacia auriculiformis* A.Cunn. ex Benth.	i	မလေးရှားပိတောက်	mələ^{3} sha^{3} bədauʔ	Malaysia-*bədauʔ*	earleaf acacia
	Albizia lebbeck (L.) Benth.	y	ကုက္ကိုပင်	kouʔ ko^{2} bin^{2}		siris tree
	Amherstia nobilis Wall.	y	သော်ကကြီး	t̪ɔ^{2} ka^{1} ji^{3}		pride of Burma
	Bauhinia acuminata L.	i	စွယ်တော်အဖြူ	swɛ^{2} dɔ^{2} əphyu^{2}		dwarf white bauhinia
	Bauhinia purpurea L.	y	စွယ်တော်	swɛ^{2} dɔ^{2}		orchid tree
	Butea monosperma (Lam.) Kuntze	y	ပေါက်ပင်	pauʔ pin^{2}		flame of the forest
	Caesalpinia pulcherrima (L.) Sw.	i	စိန်ပန်းကလေး, ဒေါင်းဆုပ်	sein^{2} ban^{3} gəle^{3}		peacock flower
	Cassia fistula L.	y	ငုပင်	ngu^{1} bin^{2}		golden shower tree, Indian laburnum
	Clitoria ternatea L.	i	အောင်မဲညို, အောင်မဲဖြူ	aun^{2} mɛ^{3} nyo^{2}, aun^{2} mɛ^{3} phyu^{2}		butterfly pea
	Delonix regia (Bojer ex Hook.) Raf.	i	စိန်ပန်း	sein^{2} ban^{3}	diamond-flower	flame tree
	Peltophorum pterocarpum (DC.) Backer ex K.Heyne	i	ပန်းမယ်ဇလီ	pan^{3} mɛ^{2} zəli^{2}	flower-*mɛzəli*	rusty shield bearer
	Pterocarpus macrocarpus Kurz, *P. indicus* Wild.	y	ပိတောက်	bədauʔ		Burma padauk, gum kino
	Samanea saman (Jacq.) Merr.	i	ကုက္ကိုပင်	kouʔ ko^{2} bin^{2}		rain tree, monkey pod tree
	Senna (Cassia) alata (L.) Roxb.	i	မဲဇလီကြီး, ပွေးကိုင်း	mɛ^{2} zəli^{2} ji^{2}, pwe^{3} gain^{3}	*mɛzəli*-big, ringworm-branch	candle bush, ringworm bush
	Sesbania grandiflora (L.) Poir.	i	ပေါက်ပန်းဖြူ, ပေါက်ပန်းနီ	pauʔ pan^{3} phyu^{2}, pauʔ pan^{3} ni^{2}		West Indian pea, vegetable hummingbird
Haemodoraceae	*Xiphidium caeruleum* Aubl.	n	ရန်ကာဂမုန်း	yan^{2} ka^{2} gəmoun^{3}		Caribbean swordleaf
Heliconiaceae	*Heliconia psittacorum* L.f.	n	ကြက်တူရွေးပန်း	ceʔ tu^{2} ywe^{3} ban^{3}	parrot-flower	parrot heliconia

	Heliconia rostrata Ruiz & Pav.	n	ဂဏန်းလက်မ	gənan^3 leʔ ma^1	crab-claw	lobster claw
Hydrangeaceae	**Hydrangea* sp.		၆လပန်း	6 la^1 pan^3	six-month-flower	hydrangea
Hypoxidaceae	*Curculigo capitulata* (Lour.) Kuntze, *C. latifolia* Dryand. ex W.T.Aiton	y	ကြွက်မလပ်	cweʔ məlaʔ		palm grass
Iridaceae	*Trimezia steyermarkii* R.C.Foster	n	ဂျပန်သစ္စာ	jəpan^2 t̪iʔ sa^2		yellow walking iris
Lamiaceae	*?Clerodendrum chinense* (Osbeck) Mabb.	y	စံပယ်ခေါင်ရမ်း	zəbɛ^2 khaun^2 yan^3		Chinese glory bower
	Clerodendrum paniculatum L.	y	?			pagoda flower
	**Clerodendrum schmidtii* C.B.Clarke	n	ဂျပန်မီးပုံး	jəpan^2 mi^3 boun^3	Japan-hot-air-balloon	chains of glory
	**Clerodendrum thomsoniae* Balf.f.	n	တိုက်ပန်း, နှစ်ပါးကြည်, ပတ္တမြားထိပ်တင်	taiʔ pan^3, hnəpa^3 ci^2, bədəmya^3 theiʔ tin^2		bleeding heart vine
	Coleus scutellarioides (L.) Benth.	y	ကတီပါရွက်လှ	gədi^2 ba^2 yweʔ hla^1	velvet-leaf-beautiful	coleus
	Ocimum tenuiflorum (sanctum) L.	y	ကုလားပင်စိမ်း	kəla^3 pin^2 zein^3	India-plant-green	holy basil
Lecythidaceae	*Couroupita guianensis* Aubl.	n	အင်ကြင်း	in^2 jin^3		cannonball tree
Lythraceae	*Lagerstroemia indica* L.	y	ပန်းအိ	pan3 i^1	flower-tender	crepe myrtle
	Lagerstroemia speciosa (L.) Martyn	y	ပျဉ်းမပင်	pyin^3 ma^1 bin^2		queen crepe myrtle
Magnoliaceae	*Magnolia* sp. (yellow)		ရေငတ်ပန်း, ဒေသရင်ခတ်	ye^2 ngaʔ pan^3, de^2 t̪a^1 yin^2 khaʔ	thirsty-flower, indigenous-*Gardenia.coronaria*	
	**Magnolia champaca* (L.) Baill. ex Pierre (yellow, white)	y	စကားဝါ, စကားဖြူ	zəgəwa^2, zəgəphyu^2	*zəga*-yellow, *zəga*-white	champak
	**Magnolia globosa* Hook.f. & Thomson	y	တတိုင်းမွှေး	ta^1 dain^3 hmwe^3	invoke-whenever-fragrant	globe magnolia
Malvaceae	**Hibiscus mutabilis* L.	n	သုံးဖန်လှ	t̪oun^3 phan^2 hla^1		changeable rose, cotton rosemallow

	Hibiscus rosa-sinensis L.	n	ခေါင်ရမ်းပန်း	khaun^{2} yan^{3} ban^{3}		hibiscus
Marantaceae	**Goeppertia (Calathea) ornata* (Lem.) Borchs. & S.Suárez	n	အပ်ချစင်း, ဒေါင်းဂမုန်း	aʔ hnəsin^{3} gəmoun^{3}, daun^{3} gəmoun^{3}	needle-two-*gəmoun*, peacock-*gəmoun*	pin-stripe calathea
Melastomataceae	**Melastoma malabathricum* L.	y	ဆေးအိုးပုတ်	hse^{3} o^{3} bouʔ		Indian rhododendron
Meliaceae	**Aglaia odorata* Lour.	n	သနပ်ခါးပင်	t̪ənəkha^{3} bin^{2}	*tanakha*.paste-flower	Chinese perfume tree
Moraceae	**Ficus elastica* Roxb. ex Hornem.	y	ညောင်ကြက်ပေါင်, ရာဘာပင်	nyaun^{2} ceʔ paun^{2}, ra^{2} ba^{2} bin^{2}	banyan-chicken.drumstick, rubber-plant	rubber plant
Muntingiaceae	*Muntingia calabura* L.	n	?			Jamaica cherry
Myrtaceae	**Melaleuca citrina* (Curtis) Dum.Cours. *(Callistemon citrinus)*	n	ပန်းပရုတ်	pan^{3} pəyouʔ	flower-balm	bottlebrush
	Syzygium fastigiatum (Blume) Merr. & L.M.Perry, *S. praetermissum* (Gage) N.P.Balakr.	y	သပြေ	d̪əbye^{2}		none
	**?Xanthostemon verdugonianus* Náves ex Fern.-Vill.	n	ပန်းမယ်ဇယ်	pan^{3} mɛ^{2} zɛ^{2}		Philippine ironwood
Nelumbonaceae	**Nelumbo nucifera* Gaertn.	y	ပဒုမ္မာကြာ	bədoun^{2} ma^{2} ca^{2}		sacred lotus
Nyctanginaceae	*Mirabilis jalapa* L.	n	၄နာရီပန်း	4 na^{2} yi^{2} pan^{3}	4-o'clock-flower	four o' clock plant
	Bougainvillea glabra Choisy (single colour, flowers sporadically)	i	စက္ကူပန်း	seʔ ku^{2} ban^{3}	paper-flower	bougainvillea
Oleaceae	**Jasminum auriculatum* Vahl	n	ဇွန်ပန်း	zun^{2} ban^{3}		Indian jasmine
	**Jasminum grandiflorum* L.	n	မုလေးပန်း, မြတ်လေးပန်း	mu^{1} le^{3} pan^{3}, myaʔ le^{3} pan^{3}		Spanish jasmine
	**Jasminum laurifolium* Roxb. ex Hornem.	y	တောစံပယ်	tɔ^{3} zəbɛ^{2}	forest-jasmine	angel-wing jasmine

	*?*Jasminum multiflorum* (Burm.f.) Andrews	y	ဇွန်စံပါယ်, တောင်စံပယ်	zun^2 zəbɛ^2, taun^2 zəbɛ^2	?June-jasmine, mountain-jasmine,	star jasmine
	Jasminum sambac (L.) Aiton	n	စံပါယ်အိုးပုတ်, ၆လ စံပါယ်, ဆောင်းစံပယ်, စပါယ်ကြီး, ၁၂ရာသီစံပါယ်	zəbɛ^2 o^3 bouʔ, 6 la^1 zəbɛ^2, hsaun^3 zəbɛ^2, zəbɛ^2 ji, 12 ya^2 dʝi^2 zəbɛ^2	jasmine-?cooking.pot, 6-month-jasmine, winter-jasmine, jasmine-big, 12-month-jasmine	Arabian jasmine
	Nyctanthes arbor-tristis L.	n	ဆိပ်ဖလူး	hseiʔ phəlu^3		parijat, night-flowering jasmine
Pandanaceae	*Pandanus tectorius (veitchii)* Parkinson	n	ဆွမ်းမွှေး	hswan^3 hmwe^3	rice.offering-fragrant	screwpine
Passifloraceae	*Passiflora coccinea* Aubl.	n	?			passionflower
	?*Turnera ulmifolia* L.	n	?			yellow alder
Plumbaginaceae	*Plumbago auriculata* Lam.	n	ကန့်ချုပ်ပြာ	kan^1 chouʔ pya^2		cape leadwort
Poaceae	*Bambusa vulgaris* Nees ‘Wamin’	y	ဝါးမင်း	wa^3 min^3	bamboo-king	Buddha belly bamboo
Polygonaceae	*Antigonon leptopus* Hook. & Arn.	n	တိုက်ပန်း	taiʔ pan^3		sandwich island creeper
Portulacaceae	*Portulaca grandiflora* Hook.	n	morning flower, ဆယ်နာရီပန်း	hsɛ^2 na^2 yi^2 pan^3	10-o’clock-flower	portulaca, rose moss
Ranunculaceae	*Clematis gouriana* Roxb. ex DC.	y	ခွါဖြူ	khwa^2 phyu^2	*khwa*-white	Indian traveller’s joy
	Clematis smilacifolia Wall.	y	ခွါညို	khwa^2 nyo^2	*khwa*-brown	tropical clematis
Rosaceae	*Rosa chinensis* Jacq.	n	နှင်းဆီ	hnin^3 zi^2		China rose
Rubiaceae	*Gardenia coronaria* Banks	y	ရင်ခတ်	yin^2 khaʔ		golden gardenia
	Gardenia jasminoides J.Ellis	n	ဇီဇဝါ, ပိန္နဲဇီဇဝါ	zi^2 zəwa^2, pein^3 nɛ^3 zi^2 zəwa^2		gardenia
	Ixora coccinea L. (red)	n	ပုဏ္ဏရိပ်	poun^2 nəyeiʔ		jungle flame, ixora

	**Mussaenda erythrophylla* Schumach. & Thonn., **M. philippica* A.Rich.	n	ပွင့်တူရွက်တူ	pwin^{1} tu ywe? tu^{2}	bloom-same-leaf-same	ashanti blood
Rutaceae	**Murraya paniculata* (L.) Jack	y	ယုဇန	yu^{1} zəna^{1}		orange jasmine/jessamine
Sapotaceae	**Mimusops elengi* L.	n	ခရေ	khəye^{2}		Spanish cherry
Solanaceae	*Cestrum nocturnum* L.	i	ညမွှေးပန်း	nya^{1} hmwe^{3} ban^{3}	night-fragrant-flower	night blooming jasmine
	Cestrum parqui (Lam.) L'Hér.	i	ညမွှေးပန်း	nya^{1} hmwe^{3} ban^{3}	night-fragrant-flower	willow-leaved jessamine
Verbenaceae	**Aloysia virgata* (Ruiz & Pav.) Juss.	n	ဆာဝါဒီခပ်	T: sa^{2} wa^{2} di^{2} khaʔ	*sawasdee kha* (Thai greeting)	sweet almond verbena
	Lantana camara L.	n	စိန်နားကပ်	sein^{2} nəgaʔ	diamond-earring	lantana
	**Petrea volubilis* L.	n	ခွာပြာ, ခင်လေးဆက်ဆက်တုန်, သမကပန်း	khwa^{2} bya^{2}, khin^{1} le^{3} hseʔ hseʔ toun^{2}, t̪əməka^{1} ban^{3}		sandpaper vine, purple wreath
Zamiaceae	**Zamioculcas zamiifolia* (G.Lodd.) Engl.	n	ကျောက်စိမ်းဂမုန်း, ကျောက်စိမ်းဧကရာဇ်	cauʔ sein^{3} gəmoun^{3}, cauʔ sein^{3} e^{2} kəriʔ	jade-*gəmoun*, jade-emperor	zamia, zz plant
Zingiberaceae	*Boesenbergia* sp.		?			fingerroot
	**Globba schomburgkii* Hook.f. (yellow)	y	ဝါဆိုပန်း	wa^{2} hso^{2} pan^{3}	*Waso*.month-flower	dancing girl ginger
	**Globba sherwoodiana* W.J.Kress & V.Gowda (white)	y	ပန်းတိမ်ငို	bədein^{2} ngo^{2}	goldsmith-cry	east-Indian swan flower
	**Hedychium coronarium* J.Koenig	y	ငွေပန်း	ngwe^{2} ban^{3}	silver-flower	white ginger lily
	**Hedychium flavum* Roxb.	y	ရွှေပန်း	shwe^{2} ban^{3}	gold-flower	yellow butterfly ginger

[1] Botanical names are 'accepted' names in POWO, elements in parentheses indicate synonyms that are still commonly used in the online horticultural trade (in Myanmar and in other countries). Cultivar names are indicative only, and are used to provide a general impression of the appearance of a plant. Non-botanical elements in parentheses describe the plant's physical appearance.

[2] Pr. denotes presence/nativeness information from POWO: y – native, i – introduced, n – not present.

[3] Burmese names are presented in abbreviated form (*i.e.* without the obligatory *pin*^{2} 'plant' element of the name) due to space constraints; see text for details.

[4] Notations used in the Transcription column: E – English origin, P – Pali, T – Thai.

Pronunciation note: A modified (simplified) version of the International Phonetic Alphabet has been used here to maximise precision and intelligibility to a wider audience. *ə*, schwa, as in the ‘a’ of ‘about’; *ɔ*, as in the ‘au’ of ‘caught’; *ɛ*, as in the ‘e’ of ‘get; *ʔ*, glottal stop; *t̪* and *d̪* indicate dental consonants; *hs*, ‘hissy s’ [s^{h}]; *hl*, *hm*, *hn*, *hng*, *hr* and *hw* are devoiced *l* [l̥], *m* [m̥], *n* [n̥], *ng* [ŋ̊], *r* [r̥] and *w* [w̥], respectively; *ph*, *th* and *kh* represent the aspirated stops [p^{h}], [t^{h}] and [k^{h}], respectively; *sh*, *ch*, *j* and *y* are pronounced as in English; *ng*, velar nasal [ŋ]; *c*, affricate [tɕ]; *n* at the end of a syllable (*e.g. oun*) indicates a nasal vowel; and superscripts 1 ‘creaky’ tone, 2 ‘low’ tone, 3 ‘high’ tone

Table S2. Newer ornamental plants that are currently being sold in physical or online stores in Yangon, Myanmar. Other details as in Table 1

Family	Latin name	Pr.	Local name	Transcription	Translation of local name	English name
Acanthaceae	*Fittonia albivenis* (Lindl. ex Veitch) Brummitt	i	မြခွက်	mya^{1} khweʔ	emerald-cup	nerve plant
	Thunbergia alata Bojer ex Sims	i	black-eyed Susan			black-eyed Susan
Amaryllidaceae	*Clivia miniata* (Lindl.) Verschaff.	n	မြေနတ်သမီး	mye^{1} naʔ t̪əmi^{3}	ground-goddess	bush lily
Annonaceae	*Mitrephora sirikitiae* Weeras., Chalermglin & R.M.K.Saunders	n	ဗြဟ္မာပန်း	byan^{2} ma^{2} pan^{3}	Brahma-flower	none
	Uvaria grandiflora Roxb. ex Hornem.	y	none			?
Apocyanaceae	*Beaumontia grandiflora* Wall.	y	နွယ်တတိုင်းမွေး	nwɛ^{2} ta^{1} dain^{3} hmwe^{3}	summer-invoke-whenever-fragrant	Easter lily vine
	Carissa carandas L.	i	ခံသီးပင်	khan^{1} d̪i^{3} pin^{2}		karonda
	Dischidia oiantha Schltr.	n	အုန်းကြား	oun^{3} ja^{3}		million hearts
	Dischidia platyphylla Schltr.	n	?			shingle plant
	Dischidia ruscifolia Decne. ex Becc.	n	ဆွဲလဲ, million heart	hswɛ^{3} lɛ^{3}		million hearts
	Dischidia vidalii Becc.	n	ငွေအိတ်ဖောင်း	ngwe^{2} eiʔ phaun^{3}	silver-bag-swollen	kangaroo pocket
	Hoya 'crimson queen'		သဲတစ်ထပ်			hoya, wax flower
	Kopsia fruticosa (Roxb.) A.DC.	y	မောင့်ချယ်ရီ	maun^{1} chɛ^{2} ri^{2}	husband-cherry	shrub vinca
	Pachypodium sp.	n	Madagascar palm			Madagascar palm
	Strophanthus caudatus (L.) Kurz	y	ပန်းဆံမိတ်	pan^{3} hsan^{2} meiʔ		climbing oleander
	Strophanthus gratus (Wall. & Hook.) Baill.	n	ဂျပန်ချယ်ရီ	jəpan^{2} chɛ^{2} ri^{2}	Japan-cherry	climbing oleander
	Wrightia antidysenterica (L.) R.Br.	n	ဂျပန်စံပယ်	jəpan^{2} zəbɛ^{2}	Japan-jasmine	Arctic snow
	Wrightia religiosa (Teijsm. & Binn.) Benth. ex Kurz	y	ကိုရီးယားဇွန်	ko^{2} ri^{3} ya^{3} zun^{2}	Korea-*zun*	water jasmine

Araceae	*Aglaonema* sp. (multicolour variegated, 'red lipstick')		အိမ်တော်မင်္ဂလာ, အိမ်တော်နီ, သနီနီ	ein^2 dɔ^2 min^2 gəla^2 gəmoun^3, ein^2 dɔ^2 ni^2 gəmoun^3, t̪əni^2 ni^2 gəmoun^3	house-royal-prosperity-*gəmoun*, house-royal-red-*gəmoun*	Chinese evergreen
	Aglaonema sp. (white variegated)		အိမ်တော်စိမ်း	ein^2 dɔ^2 sein^3 gəmoun^3	house-royal-green-*gəmoun*	Chinese evergreen
	Aglaonema sp. 'peacock'		ကျောက်စိမ်းဂမုန်း	cauʔ sein^3 gəmoun^3	jade-*gəmoun*	Chinese evergreen
	Aglaonema sp. 'pink emerald'		အန်တီနီ	an^2 ti^2 ni^2 gəmoun^3	aunty-red-*gəmoun*	Chinese evergreen
	Aglaonema sp. 'pink dalmatian', 'Chinese pink', *'China red'*		အိမ်တော်မင်္ဂလာ	ein^2 dɔ^2 min^2 gəla^2 gəmoun^3	house-royal-prosperity-*gəmoun*	Chinese evergreen
	Aglaonema sp. (red, green border)		super red			Chinese evergreen
	Aglaonema sp. (white, green border)		super white			Chinese evergreen
	Aglaonema sp. 'golden Madonna'		ပါပူးမ	pa^2 pu^3 ma^1 gəmoun^3		Chinese evergreen
	Alocasia x mortfontanensis (Alocasia x amazonica)	y				African mask plant
	Anthurium sp. (variegated)					painter's palette, flamingo flower
	Anthurium crystallinum Linden & André	n	ချမ်းသာကြီးပိန်း	chan^3 d̪a^2 ji^3 gəmoun^3	wealthy-big-*gəmoun*	crystal anthurium
	Anthurium hookeri Kunth	n	အာရက္ခံပိန်း	a^2 reʔ khan^2 gəmoun^3	?protection-*gəmoun*	bird's nest anthurium
	Colocasia esculenta (L.) Schott '?Madeira'	y	ပိန်းအနက်	pein^3 əneʔ	*pein*-black	taro

	Dieffenbachia sp. ‘big ben’		ထိုင်းမယ်တော်	thain^3 mɛ^2 dɔ^2 gəmoun^3	Thai-respected.lady-*gəmoun*	dumb cane
	Dieffenbachia cf. *seguine* (centre white, or more variegation)		ကျောက်စိမ်းဂမုန်း	cauʔ sein gəmoun^3	jade-*gəmoun*	dumb cane
	Dieffenbachia sp. ?’reflector’ (dark green, yellow spots)		ပဲလဲဖောက်, ပုလဲမင်းသား	pɛ^3 lɛ^3 phauʔ gəmoun^3, pəlɛ^3 min^3 d̪a^3 gəmoun^3	?, pearl-prince-*gəmoun*	dumb cane
	?Dieffenbachia oerstedii Schott (white midrib), ?’Sterling’	n	သော်ကကြီးဂမုန်း	t̪ɔ ka^1 ji^3 gəmoun^3		dumb cane
	?Dieffenbachia oerstedii Schott (unvariegated)	n	ထိုင်းမယ်တော်ကြီးဂမုန်း	thain^3 mɛ^2 dɔ^2 ji^3 gəmoun^3	Thai-respected.lady-*gəmoun*	dumb cane
	Epipremnum aureum (Linden & André) G.S.Bunting ‘Albo’	n	ရွှေလာငွေလာဂမုန်း	shwe^2 la^2 ngwe^2 la^2 gəmoun^3	gold-come-silver-come	golden pothos
	Monstera deliciosa Liebm. *‘Thai Constellation’*	n	ကျားလက်ဝါးဂမုန်း	ca^3 leʔ wa^3 gəmoun^3	tiger-palm-*gəmoun*	Swiss cheese plant
	?Philodendron erubescens K.Koch & Augustin	n	ဆန်းလိုက်မွန်းလိုက်ဂမုန်း	E: hsan^3 laiʔ mun^3 laiʔ gəmoun^3	sunlight-moonlight-*gəmoun*	blushing philodendron
	Philodendron ‘birkin’		ရွှေမှုန်ငွေမှုန်ဂမုန်း (ပန်းသီးဂမုန်း)	shwe^2 hmoun^2 ngwe^2 hmoun^2 gəmoun^3 (pan^3 d̪i^3 gəmoun^3)	gold-dust-silver-dust (apple-*gəmoun*)	philodendron
	Philodendron sp.		စားမကုန်သောက်မကုန်ဂမုန်း (black diamond)	sa^3 məkoun^2 t̪auʔ məkoun^2 gəmoun^3	food-inexhaustible-drink-inexhaustible-*gəmoun*	philodendron
	Philodendron sp. ‘lemon lime’		ရွှေလာဂမုန်း	shwe^2 la^2 gəmoun^3	gold-come-*gəmoun*	philodendron

	Philodendron burle-marxii G.M.Barroso 'Variegated'	n	စိန်လာဂမုန်း	sein^2 la^2 gəmoun^3	diamond-come-*gəmoun*	philodendron Burle Marx
	Philodendron longilobatum Sakur.	n	ပုရောဟိတဂမုန်း	P: pu^1 rɔ^3 hi^1 ta^1 gəmoun^3	priest-*gəmoun*	philodendron
	Philodendron opacum Croat & Grayum *(Monstera karstenianum)*	n	ပိကျူးဂမုန်း	pi^1 cu^3 gəmoun^3		philodendron
	Phyllotaenium (Caladium) lindenii André	n	မြင်းကျားဂမုန်း	myin^3 ja^3 gəmoun^3	zebra-*gəmoun*	angel's wing
	Scindapsus treubii Engl. 'moonlight'	n	ဘရာဇီးနွယ်	bəra^2 zi^3 nwɛ^2	Brazil-vine	satin pothos
	Spathiphyllum 'Jessica'	n	ရှင်သီဝလိ	shin^2 t̪i^2 wəli^1 gəmoun^3	monk.name-*gəmoun*	peace lily
Syngonium podophyllum	*Spathiphyllum* 'platinum mist'		ရှင်သီဝလိကြား	shin^2 t̪i^2 wəli^1 ca^3	monk.name-striped	peace lily
	Syngonium podophyllum Schott (pink, white variegated)	n	ရွှေလာငွေလာရွက်ကြား, စင်ကူးနီလာပန်းရောင်	shwe^2 la^2 ngwe^2 la^2 yweʔ ca^3, sin^2 ku^3 ni^2 la^2 pan^3 yaun^2	gold-come-silver-come-leaf-striped, *Syngonium*-sapphire-pink	pink syngonium, arrowhead vine
	?*Syngonium podophyllum* Schott (bluish green leaves)	n	စင်ကူး(နီးယား)နီလာ	sin^2 ku^3 (ni^3 ya^3) ni^2 la^2	*Syngonium*-sapphire	arrowhead vine
	Syngonium wendlandii Schott (white midrib, veins)	n	ကစွန်းဂမုန်း	gəzun^3 gəmoun^3	kankong-*gəmoun*	silver crowfoot
Araliaceae	*Polyscias scutellaria* (Burm.f.) Fosberg	n	ရွှေဖက်ခွက်	shwe^2 pheʔ khweʔ	gold-leaf.cup	shield aralia
Arecaceae	?*Areca triandra* Roxb. ex Buch.-Ham.	y	ကွမ်းပု	kun^3 pu^1	betel.palm-dwarf	wild areca palm
	Bismarckia nobilis Hildebrandt & H.Wendl.	n	ထန်းပြာ	than^3 bya^2	toddy-blue	
	Cyrtostachys renda Blume	n	အုံးနီ, ကွမ်းနီ	oun^3 ni^2, kun^3 ni^2	coconut-red, betel.palm-red	lipstick palm

	Hyophorbe lagenicaulis (L.H.Bailey) H.E.Moore	n	ရှန်ပိန်	E: shan^2 pein^2	champagne	bottle palm
	?Licuala grandis (T.Moore) H.Wendl.	n	စလူ	səlu^2		ruffled fan palm
	Ptychosperma propinquum (macarthurii) (Becc.) Becc. ex Martelli	n	အုန်းပွားစိမ်း	oun^3 bwa^3 sein^3	coconut-spread-green	MacArthur Palm
	Wodyetia bifurcata A.K.Irvine	n	ဖော့ဆွေး	?E: phɔ^1 hswe^3	?foxtail	foxtail palm
Asparagaceae	*Asparagus densiflorus* (Kunth) Jessop	n	none			foxtail fern
	Beaucarnea recurvata (K.Koch & Fintelm.) Lem., *?B. guatemalensis* Rose	n	ပိုနီတေး	E: po^2 ni^2 te^3	ponytail	ponytail palm
	Chlorophytum comosum (Thunb.) Jacques (middle stripe green)	n	မျက်ကြား, သဇင်မင်း	myeʔ ca^3, d̪zin^2 min^3	grass-striped, *thazin*-king	spider plant
	Cordyline fruticosa (L.) A.Chev. (red/white variegated)	i	ဇော်နီ, ဇော်မ, ဇော်မင်းသား	zɔ^2 ni^2, zɔ^2 ma^1, zɔ^2 min^3 d̪a^3		ti plant
	Dracaena angolensis (Welw. ex Carrière) Byng & Christenh. *(Sansevieria cylindrica)*	n	နဂါးစက် (နဂါးစပ်) အလုံး, ဆင်စွယ်ဂမုန်း	nəgəzeʔ (nəgəzaʔ) əloun^3, hsin^2 zwɛ^2 gəmoun^3	sansevieria-round, elephant-tusk-*gəmoun*	African spear plant
	Dracaena sanderiana Mast.	n	lucky bamboo			lucky bamboo
	Dracaena (Sansevieria) trifasciata (Prain) Mabb. 'moonshine' (broad, grey)	i	နဂါးစက် (နဂါးစပ်), snake plant	nəgəzeʔ (nəgəzaʔ)		snake plant, mother-in-law's tongue
	Dracaena (Sansevieria) trifasciata (Prain) Mabb. 'hahnii' (dwarf)	i	နဂါးစက်/နဂါးစပ်အပုမျိုး	nəgəzeʔ (nəgəzaʔ) əmyo^3		snake plant, mother-in-law's tongue
	Ophiopogon japonicus (Thunb.) Ker Gawl. 'dwarf'	n	ယိုးဒယားမျက်	yo^3 dəya^3 myeʔ	Thai-grass	mondo grass
Bignoniaceae	*Bignonia magnifica* W. Bull cf. *callistegioides* Cham.	n	နွယ်ပြာစမ်း, သူဌေးဝါဒ	nwɛ^2 pya^2 san^3, t̪əthe^3 wa^2 da^1	summer-blue-wonder,	glow vine, purple bignonia

					rich.person-ideology	
	Mansoa alliacea (Lam.) A.H.Gentry	n	ကြက်သွန်ပန်း	ceʔ t̪un² pan³	onion-flower	garlic vine
Boraginaceae	*Ehretia microphylla* Lam. *(Carmona microphylla)*	y	ကြံ့စာ, စကစ်	can¹ za², səkiʔ	rhinoceros-food	Fukien tea tree
Bromeliaceae	*Tillandsia usneoides* (L.) L.	n	မုတ်ဆိတ်	mouʔ hseiʔ	beard	Spanish moss
Caprifoliaceae	*Lonicera japonica* Thunb.	n	ရွှေတစ်ခက်ငွေတစ်ခက်ပန်း	shwe² təkhweʔ ngwe² təkhweʔ	gold-one.cup-silver-one.cup-flower	Japanese honeysuckle
Cleomaceae	*Cleome houtteana* Schltdl.	n	?			spider flower
Clusiaceae	*Clusia hilariana* Schltdl. 'variegata'	n	?			variegated autograph tree
Combretaceae	*Combretum indicum* (L.) DeFilipps	y	ယိုးဒယားထားဝယ်မှိုင်း	yo³ dəya³ dəwɛ² hmain³	Thai-Rangoon.creeper	Rangoon creeper
	Terminalia bentzoe (L.) L.f.	n	သစ်မင်းအနီ	t̪ɪʔ min³ əni²	tree-king-red	false benzoin
	Terminalia mantaly H.Perrier	n	ပန်ဒါကြာ, ထိုင်ဝမ်ဗန်ဒါ	ban² da² ca², thain² wan² ban² da²	almond-lotus, Taiwan-almond	Madagascar almond
Costaceae	*Costus woodsonii* Maas	n	နှုတ်ခမ်းနီပင်, ဖလံတောင်ဝှေး, ဂျင်းနီ	nəkhan³ ni² pan³, phəlan² taun² hwe³, jin³ ni²	lipstick-flower, caterpillar-walking.stick, ginger-red	red button ginger, scarlet spiral flag
Cupressaceae	*Cupressus* spp.	n	ဆိုက်ပရပ်, ကျောက်ခက်ပင်, ကျောက်ပန်းလုံး	E: hsaiʔ pəraʔ, cauʔ kheʔ pin², cauʔ pan³ loun³	cypress, coral-plant, ?	cypresses
Cycadaceae	*Cycas* cf. *revoluta* Thunb.	n	ဂျပန်မုန့်တိုင်	jəpan² moun² dain²		cycad
Cyperaceae	*?Carex morrowii* Boott	n	မျက်ကြား	myeʔ ca³	grass-striped	Japanese sedge
Ebenaceae	*?Diospyros vaccinioides* Lindl.	n	နှစ်၁၀၀ကြံ့စာ	hniʔ 100 can¹ za²	year-100-rhino-food	small persimmon
Equisetaceae	*Equisetum hyemale* L.	n	ရေဝါး	ye² wa³	water-bamboo	horsetail rush

Euphorbiaceae	*Codiaeum variegatum* (L.) Rumph. ex A.Juss. (yellow-green, red underside)	n	ရွက်လှ-စွန်ရဲ	ywe? hla^{1} – sun^{2} yɛ^{3}	leaf-beautiful – kite-red	croton
Fabaceae	*Barnebydendron riedelii* (Tul.) J.H.Kirkbr. *(Phyllocarpus septentrionalis)*	n	none			monkey flower tree
	Brownea grandiceps Jacq.	i	ကိုလံဘီယာသော်ကကြီး	ko^{2} lan^{2} bi^{2} ya^{2} t̪ɔ^{2} ka^{1} ji^{3}	Columbia-*Amherstia*	rose of Venezuela
	Cassia javanica L.	y	ငုစပ်ချယ်ရီ	ngu^{1} sa? chɛ^{2} ri^{2}	*Cassia.fistula*-hybrid-cherry	Java cassia
	Christia vespertilionis (L.f.) Bakh.f.	i	လိပ်ပြာပန်း	lei? pya^{2} pan^{3}	butterfly-flower	red butterfly wing
	Clitoria arborea Benth.	n	ငုပြာ	ngu^{1} pya^{2}	*Cassia.fistula*-blue	butterfly pea tree
	Dendrolobium umbellatum (L.) Benth.	y	ရွှေသစ်ရွက်	shwe^{2} t̪i? ywe?	gold-leaf	horse bush
	Erythrina crista-galli L.	n	အစ္စရေးကသစ်	i? səre^{3} kət̪i?	Israel-*Erythrina*	cockspur coral tree
	Phyllodium pulchellum (L.) Desv.	y	ငွေဆွဲလည်း, ငွေဆည်းလည်း, ဒင်္ဂါး	ngwe^{2} hswɛ^{3} lɛ^{3}, ngwe^{2} hsɛ^{3} lɛ^{3}, din^{3} ga^{3}	silver-small.bell, coin	
	Pterocarpus santalinus L.f.	n	နံ့သာနီ	nan^{1} t̪a^{2} yi^{2}	smell-pleasant-red	red sandalwood
	Wisteria cf. *sinensis* (Sims) DC.	n	none			Chinese wisteria
Gentianaceae	*Cyrtophyllum (Fagraea) fragrans* (Roxb.) DC.	y	အနမ်းပန်း	ənan^{3} ban^{3}		tembusu
	Fagraea auriculata Jack	y	အနမ်းပန်း	ənan^{3} ban^{3}		?
	Fagraea ceilanica Thunb.	y	none			perfume flower tree
Gesneriaceae	*Episcia cupreata* (Hook.) Hanst.	n				flame violet
Lamiaceae	*Coleus scutellarioides* (L.) Benth. (small leaved, yellow border)	y	ပါတိတ်ရွက်လှ	pa^{2} tei? ywe? hla^{1}	batik-leaf-beautiful	coleus
	Petraeovitex bambusetorum King & Gamble	n	နွဲ့နှောင်း	nwɛ^{1} hnaun^{3}		nong noch vine
Lecythidaceae	*Barringtonia racemosa* (L.) Spreng.	y	ညနတ်သမီး	nya^{1} na? t̪ami^{3}	night-goddess	powderpuff mangrove
	Gustavia cf. *superba* (Kunth) O.Berg	n	နိဗ္ဗာန်ကြာ	nei? ban^{2} ca^{2}	nirvana-lotus	heaven lotus

Lythraceae	*Cuphea hyssopifolia* Kunth	n	စတားဝါး, ကြယ်တာရာ	E: səta^3 wa^3, cɛ^2 ta^2 ya^2	star wars, constellation	false heather
Magnoliaceae	*Magnolia compressa* Maxim.	n	စစ်ခွန်းသာ/စဝ်ခွန်းသာ	siʔ khun^3 t̪a^2, saʔ khun^3 t̪a^2		Formosan michelia
	Magnolia liliiflora Desr.	n	စကားနီ	zəgəni^2	*zəga*-red	
	Magnolia figo (Lour.) DC.	n	ဂျပန်တတိုင်းမွှေး အနီ/အဖြူ	jəpan^2 ta^1 dain^3 hmwe^3 əni^2/ əphyu^2	Japan-invoke-whenever-fragrant-red/ white	banana shrub
	Magnolia kobus DC.	n	ရေငတ်ပန်း/ဒေသရင်ခတ်	ye^2 ngaʔ pan^3, de^2 t̪a^1 yin^2 khaʔ	thirsty-flower, indigenous-*Gardenia.coronaria*	northern Japanese magnolia
Malpighiaceae	*Galphimia gracilis* Bartl.	n	ဂျပန်ပိတောက်	jəpan^2 bədauʔ	Japan-*padauk*	shower of gold
	Lophanthera lactescens Ducke	n	ရွှေချိန်း	E: shwe^2 chein^3	gold-chain	golden chain tree
	Tristellateia australasiae A.Rich.	y				showers of gold climber
Malvaceae	*?Heritiera littoralis* Aiton	y	နတ်မွှေးပန်း	naʔ hmwe^3 pan^3	spirit-fragrant-flower	looking glass tree
	Pachira aquatica Aubl.	n	ထီပေါက်ပင်	thi^2 pauʔ pin^2	lottery-win-plant	money tree
Marantaceae	*Calathea* sp. (purple leaves)					calathea, prayer plant
	Ctenanthe setosa (Roscoe) Eichler 'grey star'	n	ထံရံကပ်ဂမုန်း	thəyan^2 kaʔ gəmoun^3	wall-stick-*gəmoun*	never never plant, ctenanthe
	Ctenanthe burle-marxii H.Kenn.	n	ဒေါင်းမြီးရွက်ဂမုန်း, ကျီးခြေဂမုန်း	daun^3 mi^3 yweʔ gəmoun^3, ci^3 che^2 gəmoun^3	peacock-tail-leaf-*gəmoun*, crow-foot-*gəmoun*	never never plant, ctenanthe
	Goeppertia insignis (W.Bull ex W.E.Marshall) J.M.A.Braga, L.J.T.Cardoso & R.Couto	n	ဣစ္ဆာသရကျီးမင်းဂမုန်း	iʔ sa^2 t̪əra^1 ci^3 min^3 gəmoun^3	cornucopia-crow-king-*gəmoun*	rattlesnake plant
	Goeppertia kegeljanii (É.Morren) Saka *(Calathea musaica)*	n	ထံရံကပ်ဂမုန်း	thəyan^2 kaʔ gəmoun^3	wall-stick-*gəmoun*	calathea, prayer plant

	Goeppertia (Calathea) loeseneri (J.F.Macbr.) Borchs. & S.Suárez 'crimson'	n	ကုန်းကြာဂမုန်း	koun^{3} ca^{2} gəmoun^{3}	land-lotus-*gəmoun*	calathea, prayer plant
	Goeppertia makoyana (É.Morren) Borchs. & S.Suárez	n				peacock calathea
	Goeppertia (Calathea) orbifolia (Linden) Borchs. & S.Suárez	n	ပန်းသီးဂမုန်း	pan^{3} d̪i^{3} gəmoun^{3}	apple-*gəmoun*	calathea, prayer plant
	Goeppertia picturata (K.Koch & Linden) Borchs. & S.Suárez	n	ဒေါင်းမင်းဂမုန်း	daun^{3} min^{3} gəmoun^{3}	peacock-king-*gəmoun*	calathea, prayer plant
	Goeppertia (Calathea) roseopicta (Linden ex Lem.) Borchs. & S.Suárez	n				calathea, prayer plant
	Stromanthe thalia (sanguinea) 'Tricolor' (Vell.) J.M.A.Braga	n	ရှင်ချုပ်ဂမုန်း	shin^{2} chouʔ gəmoun^{3}		stromanthe
Moraceae	*Dorstenia elata* Gardner	n	none			Congo fig
	Ficus benjamina L. (variegated)	y	ညောင်ကြား	nyaun^{2} ja^{3}	banyan-striped	weeping fig
	Ficus lyrata Warb.	n	စင်ကာပူဗန်ဒါ	sin^{2} ga^{2} pu^{2} ban^{2} da^{2}	Singapore-almond	fiddle-leaf fig
	Ficus microcarpa L.f. 'panda'	y	Mexico ညောင်, ဘုံသုံးဆင့်	Mexico nyaun^{2}, boun^{2} t̪oun^{3} hsin^{1}	Mexico-banyan, plane.of.existence-three-level	Chinese banyan
	Ficus natalensis subsp. leprieurii (Miq.) C.C.Berg *(F. triangularis)* 'Variegata'	n	ညောင်ယပ်တောင်, အသဲညောင်	nyaun^{2} yaʔ tɔ^{2}, ət̪ɛ^{3} nyaun^{2}	banyan-fan, liver-banyan	triangle fig
Myrtaceae	*?Melaleuca linariifolia* Sm.	n	ပရုတ်မွှေးပန်း	pəyouʔ hmwe^{3} pan^{3}	balm-fragrant-flower	honey myrtle
	?Psidium guajava L. 'Nigrum'	y	ပန်းသီးမာလကာ	pan^{3} d̪i^{3} ma^{2} ləka^{2}	apple-guava	black guava
	?Syzygium myrtifolium Walp.	y	ပတ္တမြားသပြေ, ရွှေသပြေ, အာစီယံသပြေ	bədəmya^{3} d̪əbye^{2}, shwe^{2} d̪əbye^{2}, a^{2} si^{2} yan^{2} d̪əbye^{2}	ruby-*Syzygium*, gold-*Syzygium*, ASEAN-*Syzygium*	red lip

	?Syzygium salicifolium J.Graham	n	စိန်တဆုပ်	sein^2 təhsouʔ	diamond-fistful	?
	Xanthostemon youngii C.T.White & W.D.Francis, *?X. chrysanthus* (F.Muell.) Benth.	n	ယိုးဒယားပန်းမယ်ဇယ်	yo^3 dəya^3 pan^3 mɛ^2 zɛ^2	Thai-*?Xanthostemon*	crimson/yellow penda
Nyctanginaceae	*Bougainvillea glabra* Choisy (multiple colours, flowers en masse)	i	စက္ကူပန်း, အိန္ဒိယစက္ကူပန်း	seʔ ku^2 ban^3, ein^2 di^1 ya^1 seʔ ku^2 ban^3	paper-flower, India-paper-flower	bougainvillea
Oxalidaceae	*Oxalis triangularis* A.St.-Hil.	n	လိပ်ပြာပန်း	leiʔ pya^2 ban^3	butterfly-flower	false shamrock
Passifloraceae	*Passiflora* spp. (red flower)		?			passion flower
	Turnera ulmifolia L.	n	?			yellow alder
Piperaceae	*Peperomia* 'Watermelon'		ဖရဲဂမုန်း	phəyɛ^3 gəmoun^3	watermelon-*gəmoun*	watermelon peperomia
	Peperomia caperata Yunck.	n	ငွေဖရဲရွက်လှ	ngwe^2 phəyɛ^3 yweʔ hla^1	silver-watermelon-leaf-beautiful	
Poaceae	*Axonopus compressus* (Sw.) P.Beauv. (variegated)	i	ယိုးဒယားမြက်ကြား	yo^3 dəya^3 myeʔ ca^3	Thai-grass-striped	variegated pearl grass
	?Phalaris arundinacea L.	y	မျက်ကြား	myeʔ ca^3	grass-striped	reed canary grass
Podocarpaceae	*Podocarpus macrophyllus* (Thunb.) Sweet	y	ယိုးဒယားသစ်မင်း, ထိုင်ဝမ်သစ်မင်း	yo^3 dəya^3 ţiʔ min^3, thain^2 wan^2 ţiʔ min^3	Thai-tree-king, Taiwan-tree-king	yew plum pine
	Podocarpus neriifolius D.Don	y	သစ်မင်းဖို	ţiʔ min^3 pho^2	tree-king-male	
Polypodiaceae	*?Microsorum punctatum* (L.) Copel.	y	ဆိတ်ဂျို	hseiʔ jo^2	goat-horn	elkhorn fern
	Nephrolepis exaltata (L.) Schott	n	ငါးရိုး fern	nga^3 yo^3 *fern*	fish-bone-fern	Boston fern
Primulaceae	*Ardisia elliptica* Thunb.	y	ခရေနီ	khəye^2 ni^2	*Mimusops*-red	
Pteridaceae	*Adiantum* sp.					maidenhair fern
Rosaceae	*Spiraea cantoniensis* Lour.	n	none			Reeves bridal wreath spirea
Rubiaceae	*Arachnothryx leucophylla* (Kunth) Planch.	n	ညမွှေးပန်းပန်းရောင်, ယိုးဒရားညမွှေးပန်း	nya^1 hmwe^3 ban^3 pan^3 yaun^2, yo^3	night-fragrant-flower-pink, Thai-	Panama rose

				dəya^3 nya^1 hmwe^3 ban^3	night-fragrant-flower	
	Cephalanthus tetrandrus (occidentalis) (Roxb.) Ridsdale & Bakh.f.	y	ဘောလုံးလေး	bɔ^3 loun^3 ban^3	ball-flower	buttonbush
	Gardenia jasminoides J.Ellis cultivar	n	ဗီယက်နမ်ဇီဇဝါ	bi^2 yeʔ nam^2 zi^2 zəwa^2	Vietnam-gardenia	Cape jasmine
	Gardenia scabrella Puttock	n	ရင်ခတ်ဖြူ	yin^2 khaʔ phyu^2	*Gardenia.coronaria*-white	star flower
	Ixora sp. (dwarf, yellow, orange, pink, white, variegated)		ပုဏ္ဏရိပ်, Mexico bonsai ပုဏ္ဏရိပ်	poun^2 nəyeiʔ, Mexico bonsai poun^2 nəyeiʔ		ixora
	Ixora 'Dwarf white'		ဆန်ကွဲပန်း	zəgwɛ^3 pan^3	broken.rice-flower	dwarf white ixora
	Pentas lanceolata	n	ပန်းသက်ရှည်	pan^3 t̪ɛʔ she^2	flower-life-long	Egyptian starcluster
	Tarenna fragrans (Forssk.) Deflers	n	ဘုံခုနစ်ဆင့်မွှေး	boun^2 7 hsin^1 hmwe^3	plane.of.existence-seven-level-fragrant	?
Rutaceae	*Murraya paniculata* (L.) Jack (large leafed)	y	ယုဇနအရွက်ကြီး	yu^1 zəna^1 əyweʔ ci^3	*Gardenia*-leaf-big	orange jasmine/jessamine
Salicaceae	*Oncoba spinosa* Forssk.	n	စူးကံ့ကော်	su^3 gan^1 gɔ^2	thorn-*Mesua.ferrea*	fried egg tree
	Salix babylonica L.	n	မိုးမခပင်	mo^3 məkha^1 bin^2		weeping willow
Solanaceae	*Brunfelsia pauciflora* (Cham. & Schltdl.) Benth.	n	ဖြူပြာမူယာ	phyu^2 pya^2 mu^2 ya^2	blue-white-expression	yesterday today and tomorrow
Theaceae	*Camellia azalea* C.F.Wei	n	လက်ဖက်နှင်းဆီ	ləpheʔ hnin^3 zi^2	tea-rose	none
Urticaceae	*Pilea cadierei* Gagnep. & Guillaumin	n				aluminium plant
Verbenaceae	*Citharexylum flexuosum* (Ruiz & Pav.) D.Don	n	တော်ဝင်သဇင်	tɔ^2 win^2 dəz̪in^2	fit.for.royalty-*thazin*	fiddlewood
Zingiberaceae	*Alpinia purpurata* (Vieill.) K.Schum.	n	red ginger			red ginger
	Globba winitii C.H.Wright (purple)	y	ပန်းတိမ်ငို ခရမ်း	bədein^2 ngo^2 khəyan^3	goldsmith-cry-purple	dancing lady ginger
	?*Kaempferia* sp.					peacock ginger

Zygophyllaceae	*Guaiacum officinale* L.	n	ယုဇနအပြာရောင်	yu^{1} zəna^{1} əpya^{2} yaun^{2}	*Murraya*-blue	roughbark lignum-vitae

Table S3. New plant names based on pre-existing ones (the latter shown in bold for each group). Groups presented here share a 'generic' label, and also contain at least one old and one new ornamental plant. In the Old/New column, o indicates 'old', *i.e.* an ethnotaxon that has been cultivated in Yangon since the 1980s/1990s, while n indicates 'new', *i.e.* a recent arrival.

Local name	Local name translation	Latin name (Family)	Old/New
**t̪i? min^{3}**		*Yucca* sp. (Asparagaceae)	o
t̪i? min^{3} əni^{2}	*t̪i?min^{3}*-red	*Terminalia bentzoe* (Combretaceae)	n
yo^{3} dəya^{3} t̪i? min^{3}, thain^{2} wan^{2} t̪i? min^{3}	Thai- *t̪i?min^{3}*, Taiwan- *t̪i?min^{3}*	*Podocarpus macrophyllus* (Podocarpaceae)	n
t̪i? min^{3} pho^{2}	*t̪i?min^{3}*-male	*Podocarpus neriifolius* (Podocarpaceae)	n
**t̪ɔ^{2} ka^{1} dədain^{3}**		*Pyrostegia venusta* (Begoniaceae)	o
t̪ɔ^{2} ka^{1} ji^{3}	*t̪ɔ^{2}ka^{1}*-big	*Amherstia nobilis* (Fabaceae)	o
t̪ɔ ka^{1} ji3 gəmoun^{3}	*t̪ɔ^{2}ka^{1}*-big-*gəmoun*	*?Dieffenbachia oerstedii* (white midrib), ?'Sterling' (Araceae)	n
ko^{2} lan^{2} bi^{2} ya^{2} t̪ɔ^{2} ka^{1} ji^{3}	Columbia-*t̪ɔ^{2}ka^{1}*-big	*Brownea grandiceps* (Fabaceae)	n
**bədau?**		*Pterocarpus macrocarpus, P. indicus* (Fabaceae)	o
bədau? ni^{2}	*bədau?*-red	*Radermachera sinica* (Begoniaceae)	o
məle^{3} sha^{3} bədau?	Malaysia-*bədau?*	*Acacia auriculiformis* (Fabaceae)	o
jəpan^{2} bədau?	Japan-*bədau?*	*Galphimia gracilis* (Malpighiaceae)	n
**gan^{1} gɔ^{2}**		*Mesua ferrea* (Calophyllaceae)	o
su^{3} gan^{1} gɔ^{2}	thorn-*gan^{1}gɔ^{2}*	*Oncoba spinosa* (Salicaceae)	n
**zɔ^{2} ji^{2} mou? hsei?**		*Tacca chantrieri* (Dioscoreaceae)	o
zɔ2 ji^{2} mou? sei? t̪i? khwa^{1}	*zɔ^{2}ji^{2}mou?hsei?*-orchid	*Paphiopedilum parishii* (Orchidaceae)	n
**d̪əbye^{2}**		*Syzygium fastigiatum, Syzygium praetermissum* (Myrtaceae)	o
bədəmya^{3} d̪əbye^{2}, shwe^{2} d̪əbye^{2}, a^{2} si^{2} yan^{2} d̪əbye^{2}	ruby-*d̪əbye^{2}*, gold- *d̪əbye^{2}*, ASEAN-*d̪əbye^{2}*	*?Syzygium myrtifolium* (Myrtaceae)	n
**zun^{2} ban^{3}**		*Jasminum auriculatum* (Oleaceae)	o
ko^{2} ri^{3} ya^{3} zun^{2}	Korea-*zun^{2}*	*Wrightia religiosa* (Apocyanaceae)	n
**zəbɛ^{2}**		*Jasminum sambac* (Oleaceae)	o

tɔ^{3} zəbɛ^{2}	forest-*zəbɛ^{2}*	*Jasminum laurifolium* (Oleaceae)	o
taun^{2} zəbɛ^{2}	mountain- *zəbɛ^{2}*	*?Jasminum multiflorum* (Oleaceae)	o
jəpan^{2} zəbɛ^{2}	Japan-*zəbɛ^{2}*	*Wrightia antidysenterica* (Apocyanaceae)	n
**hnin^{3} zi^{2}**		*Rosa chinensis* (Rosaceae)	o
gan^{2} da^{2} ya^{1} hnin^{3} zi^{2}	desert-*hnin^{3}zi^{2}*	*Adenium obesum* (Apocyanaceae)	o
ləpheʔ hnin^{3} zi^{2}	tea-*hnin^{3}zi^{2}*	*Camellia azalea* (Theaceae)	n
**zi^{2} zəwa^{2}**		*Gardenia jasminoides* (Rubiaceae)	o
bi^{2} yeʔ nam^{2} zi^{2} zəwa^{2}	Vietnam-*zi^{2}zəwa^{2}*	*Gardenia jasminoides* cultivar (Rubiaceae)	n
**yu^{1} zəna^{1}**		*Murraya paniculata* (Rutaceae)	o
yu^{1} zəna^{1} əpya^{2} yaun^{2}	*yu^{1}zəna^{1}*-blue	*Guaiacum officinale* (Zygophyllaceae)	n
**d̪əzin^{2} ban^{3}**		*Bulbophyllum auricomum* (Orchidaceae)	o
d̪əzin^{2} min^{3}	*d̪əzin^{2}*-king	*Chlorophytum comosum* (Asparagaceae)	n
tɔ^{2} win^{2} d̪əzin^{2}	fit.for.royalty-*d̪əzin^{2}*	*Citharexylum flexuosum* (Verbenaceae)	n
d̪əzin^{2} cɛ^{2} t̪iʔ khwa^{1}	*d̪əzin^{2}*-star-orchid	*Bulbophyllum lobbii* subsp. *siamense* (Orchidaceae)	n
d̪əzin^{2} ji^{3} əphyu^{2} t̪iʔ khwa^{1}	*d̪əzin^{2}*-big-white-orchid	*Calanthe triplicata* (Orchidaceae)	n
**khəye^{2}**		*Mimusops elengi* (Sapotaceae)	o
khəye^{2} ni^{2}	*khəye^{2}*-red	*Ardisia elliptica* (Primulaceae)	n
**nya^{1} hmwe^{3} ban^{3}**		*Cestrum nocturnum, C. parqui* (Solanaceae)	o
nya^{1} hmwe^{3} ban^{3} pan^{3} yaun^{2}, yo^{3} dəya^{3} nya^{1} hmwe^{3} ban^{3}	*nya^{1}hmwe^{3}ban^{3}*-pink, Thai-*nya^{1}hmwe^{3}ban^{3}*	*Arachnothryx leucophylla* (Rubiaceae)	n
**wa^{2} hso^{2} pan^{3}**		*Globba schomburgkii* (Zingiberaceae)	o
wa2 hso2 pan3 t̪iʔ khwa1	*wa^{2}hso^{2}pan^{3}*-orchid	*Dendrobium moschatum* (Orchidaceae)	n
**bədein^{2} ngo^{2}**		*Globba sherwoodiana* (Zingiberaceae)	o
bədein^{2} ngo^{2} khəyan^{3}	*bədein^{2}ngo^{2}khəyan^{3}*-purple	*Globba wintii* (Zingiberaceae)	n

Table S4. Older and newer orchid varieties grown in Yangon, Myanmar. The newer orchid names were sourced mainly from online sellers (on Facebook) and from [27–29].

Older					
Latin name	Local name	Transcription	Translation of local name	English name	Pr.
Arachnis flos-aeris (L.) Rchb.f., *A.* x *maingayi* (Hook.f.) Schltr.	spider			spider orchid	n, y
Bulbophyllum auricomum Lindl.	သဇင်ပန်း	d̪əzin^{2} ban^{3}	*thazin*-flower	thazin orchid	y
Calanthe (Phaius) tankervilleae (Banks) M.W.Chase, Christenh. & Schuit.	မောင်ကြီးပက်လက်လန်	maun^{2} ji^{3} peʔ leʔ lan^{2}	MAN.TITLE-on.back-lying	nun's hood orchid	y
Cattleya labiata Lindl.	cattleya			cattleya	n
Cleisostoma simondii (Gagnep.) Seidenf.	တုတ်ချောင်းသစ်ခွ, အရိုးသစ်ခွ	douʔ chaun^{3} t̪iʔ khwa^{1}, əyo t̪iʔ khwa^{1}	stick-orchid	stick orchid	y
Cymbidium aloifolium (L.) Sw.	သစ်တက်လင်းနေ	t̪iʔ teʔ lin^{3} ne^{2}	tree-climb-shine-sun		y
?Cyrtochilum (Oncidium) flexuosum x sphacelatum	ဒန်းဇင်း	E: dan^{3} zin^{3}	dancing	dancing lady orchid	n
Dendrobium aphyllum (Roxb.) C.E.C.Fisch.	လက်တံရှည်သစ်ခွ	leʔ tan^{2} she^{2} t̪iʔ khwa^{1}	arm-long-orchid	hooded orchid	y
Dendrobium chrysanthum Wall. ex Lindl.	ရွှေတဆုပ်သစ်ခွ	shwe^{2} təhsouʔ t̪iʔ khwa^{1}	gold-fistful-orchid	golden-flowered Dendrobium	y
Dendrobium densiflorum Lindl.	တစ်ခိုင်လုံးရွှေသစ်ခွ	təkhain^{2} loun^{3} t̪iʔ khwa^{1}	branch-entire-orchid	pineapple orchid	y
Dendrobium farmeri Paxton	ငွေတူကလေးသစ်ခွ	ngwe^{2} tu^{2} gəle^{3} t̪iʔ khwa^{1}	silver-similar-little-orchid		y
Dendrobium fimbriatum Hook.	လက်တံရှည်အာမဲသစ်ခွ	leʔ tan^{2} she^{2} a^{2} mɛ^{3} t̪iʔ khwa^{1}	arm-long-black-orchid	fringe-lipped Dendrobium	y
Dendrobium findlayanum C.S.P.Parish & Rchb.f.	ချိုချဉ်သစ်ခွ	cho^{2} chin^{2} t̪iʔ khwa^{1}	sweet-sour-orchid		y
Dendrobium formosum Roxb. ex Lindl.	ငွေပန်းသစ်ခွ	ngwe^{2} ban^{3} t̪iʔ khwa^{1}	silver-flower-orchid		y
Dendrobium lindleyi Steud.	ရတနာရွှေခက်သစ်ခွ	yədəna^{2} shwe^{2} kheʔ t̪iʔ khwa^{1}	treasure-gold-flower.branch-orchid		y

Dendrobium pulchellum Rolfe cf. *loddigesii* Rolfe	ဆင်မမျက်ကွင်းသစ်ခွ	hsin² ma¹ myeʔ kwin³ t̪iʔ khwa¹	elephant-female-eye-orchid	charming Dendrobium	y
Epidendrum sp.	ချင်းလုံးသစ်ခွ	chin³ loun³ t̪iʔ khwa¹	rattan.ball-orchid		y
Papilionanthe teres (Roxb.) Schltr.	တုတ်ချောင်းသစ်ခွ, အရိုးသစ်ခွ	douʔ chaun³ t̪iʔ khwa¹, əyo³ t̪iʔ khwa¹	stick-orchid	stick orchid	y
Rhynchostylis (Aerides) retusa (L.) Blume, *?R. gigantea* (Lindl.) Ridl.	ကြောင်မြီးသစ်ခွ	caun² hmi³ t̪iʔ khwa¹	cat-tail-orchid	foxtail orchid	y
Spathoglottis plicata Blume (deep pink)	အုန်းသစ်ခွ	oun³ t̪iʔ khwa¹	coconut.palm-orchid	Philippine ground orchid	y
Vanda coerulea Griff. ex Lindl.	မိုးလုံးမှိုင်းသစ်ခွ	mo³ loun³ hmain³ t̪iʔ khwa¹	sky-all-hazy-orchid	blue Vanda	y
Newer					
Latin name	Local name	Transcription	Translation of local name	English name	Pr
Aerides falcata Lindl. & Paxton	စာကလေးသစ်ခွ	sa² gəle³ t̪iʔ khwa¹	sparrow-orchid		y
Aerides spp.	ယိုးဒယားစာကလေးစပ်	yo³ dəya³ sa² gəle² saʔ	Thai-sparrow-hybrid		y
Bulbophyllum lobbii subsp. *siamense* (Rchb.f.) Mangal, F.Velazquez & J.J.Verm.	သဇင်ကြယ်သစ်ခွ	d̪əzin² cɛ² t̪iʔ khwa¹	*thazin*-star-orchid		y
Calanthe triplicata (Willemet) Ames	သဇင်ကြီးအဖြူသစ်ခွ	d̪əzin² ji³ əphyu² t̪iʔ khwa¹	*thazin*-big-white-orchid	Christmas orchid	y
Cleisostoma discolor Lindl., *C. crochetii* (Guillaumin) Garay	ကြွက်မြီးသစ်ခွ	cweʔ hmi³ t̪iʔ khwa¹	rat-tail-orchid		n, y
Coelogyne sp.	ငွေနှင်းဖြူသစ်ခွ	ngwe² hnin³ phyu² t̪iʔ khwa¹	silver-snow-white-orchid		y
Cymbidium spp.	ပန်းသက်ရှည်	pan³ t̪eʔ she²	flower-life-long	boat orchid	
Dendrobium anosmum Lindl.	ခရမ်းမွှေးသစ်ခွ	khəyan³ hmwe³ t̪iʔ khwa¹	purple-fragrant-orchid	unscented Dendrobium	y
Dendrobium brymerianum Rchb.f.	ရွှေမျှင်, ရွှေကနုတ်သစ်ခွ	shwe² hmyin², shwe² kənouʔ t̪iʔ khwa¹	silver-thread, golden-floral.carving-orchid		y
Dendrobium capillipes Rchb.f.	ဖယောင်းခြေတိုသစ်ခွ	phəyaun³ che² to² t̪iʔ khwa¹	wax-leg-short-orchid		y
Dendrobium cariniferum Rchb.f.	မဟာဒေဝီသစ်ခွ	məha² de² wi² t̪iʔ khwa¹	great-lady-orchid		y

Dendrobium Chaisri Gold	ယုန်နားရွက်	youn^{2} na^{3} ʈʝʔ khwa^{1}	rabbit-ear-orchid		y
Dendrobium fimbriatum Hook. cf. *chryseum* Rolfe	မောက်ခမ်းဝါ(အာမဲ)	mauʔ khan^{3} wa^{2} (a^{2} mɛ^{3})	crest-lip-yellow (throat-black)		y
Dendrobium gratiosissimum Rchb.f.	နန်းမာလာသစ်ခွ	nan^{3} ma^{2} la^{2} ʈʝʔ khwa^{1}			y
Dendrobium moschatum (Banks) Sw.	ဇလပ်ထော်သစ်ခွ, ဝါဆိုပန်းသစ်ခွ	zəlaʔ thɔ^{2} ʈʝʔ khwa^{1}, wa^{2} hso^{2} pan^{3} ʈʝʔ khwa^{1}	*Tabernaemontana*-pout-orchid, *waso*.month-flower-orchid		y
Dendrobium nobile Lindl.	ဒေါင်းမြီးသစ်ခွ	daun^{3} hmi^{3} ʈʝʔ khwa^{1}	peacock-tail-orchid		y
Dendrobium polyanthum (cretaceum) Wall. ex Lindl.	ခေါင်းလောင်းဖြူသစ်ခွ	khaun^{3} laun^{3} phyu^{2} ʈʝʔ khwa^{1}	bell-white-orchid		y
Dendrobium primulinum Lindl.	သင်းကြူကြူသစ်ခွ	ʈʝn^{3} cu^{2} cu^{2} ʈʝʔ khwa^{1}	mild-fragrantly-orchid	primrose yellow Dendrobium	y
Dendrobium secundum (Blume) Lindl. ex Wall.	သွားပွတ်တံသစ်ခွ	dʝəbuʔ tan^{2} ʈʝʔ khwa^{1}	toothbrush-orchid	toothbrush orchid	y
Dendrobium sp.	ယာယာလျှာပြား, လျှာပြားအဖြူ	ya^{2} ya^{2} sha^{2} bya^{3}, sha^{2} bya^{3} əphyu^{2}	?, tongue-flat-white		y
Dendrobium sutepense Rolfe ex Downie	စံပယ်သစ်ခွ	zəbɛ^{2} ʈʝʔ khwa^{1}	jasmine-orchid		y
Dendrobium tortile Lindl.	သူယောင်သစ်ခွ	ʈʝu^{2} yaun^{2} ʈʝʔ khwa^{1}	mythical.vine-orchid	twisted Dendrobium	y
Dendrobium transparens Wall. ex Lindl.	မနှင်းဖြူသစ်ခွ	maʔ hnin^{3} phyu^{2} ʈʝʔ khwa^{1}	Miss-snow-white-orchid		y
Gastrochilus calceolaris (Buch.-Ham. ex Sm.) D.Don	ပျားအုံသစ်ခွ	pya^{3} oun^{2} ʈʝʔ khwa^{1}	bee-hive-orchid		y
Grammatophyllum speciosum Blume	ကျားဗဟုန်းသစ်ခွ, သစ်ခွဘုရင်မ	ca^{3} ba^{1} houn^{3} ʈʝʔ khwa^{1}, ʈʝʔ khwa^{1} bəyin^{2} ma^{1}	?, orchid-queen	giant orchid, tiger orchid	y
Paphiopedilum bellatulum (Rchb.f.) Stein	ခွန်မြစမ်း, ငုံးဥသစ်ခွ	khun^{2} mya^{1} san^{3}, ngoun^{3} u^{1} ʈʝʔ khwa^{1}	?, quail-egg-orchid	egg-in-a-nest orchid	y
Paphiopedilum parishii (Rchb.f.) Stein	ဇော်ဂျီမုတ်ဆိတ်သစ်ခွ	zɔ^{2} ji^{2} mouʔ seiʔ ʈʝʔ khwa^{1}	mythical.being-beard-orchid		y
Paphiopedilum spicerianum (Rchb.f.) Pfitzer	မြွေမင်းသမီးသစ်ခွ	mwe^{2} min^{3} dʝəmi^{3} ʈʝʔ khwa^{1}	snake-princess-orchid		y

Papilionanda T.M.A.	TMA			papilionanda	n
Phalaenopsis marriottiana (Rchb.f.) Kocyan & Schuit. *(Hygrochilus parishii)*	တောင်ကရမက်သစ်ခွ	taun^{2} kərəmeʔ t̪iʔ khwa^{1}	mountain-false.sandalwood-orchid		y
Phalaenopsis spp.	လိပ်ပြာသစ်ခွ	leiʔ pya^{2} t̪iʔ khwa^{1}	butterfly-orchid	moth orchid	y
Pinalia (Eria) amica (Rchb.f.) Kuntze	နတ်သမီးသစ်ခွ	naʔ t̪əmi t̪iʔ khwa^{1}	female.spirit-orchid		y
Spathoglottis plicata Blume (various colours)	အုန်းသစ်ခွ	oun^{3} t̪iʔ khwa^{1}	coconut.palm-orchid	Philippine ground orchid	y
Thunia alba (Lindl.) Rchb.f.	ကျောက်သစ်ခွ	cauʔ t̪iʔ khwa^{1}	rock-orchid		y
Vanda bensonii Bateman	မိုးသူဇာသစ်ခွ	mo^{3} t̪u^{2} za^{2} t̪iʔ khwa^{1}	FEMALE.NAME-orchid		y
Vanda curvifolia (Lindl.) L.M.Gardiner *(Ascocentrum curvifolium)*	သန္တာလေးနီသစ်ခွ	t̪an^{2} da^{2} le^{3} ni^{2} t̪iʔ khwa^{1}	coral-little-orchid		y
Oncidium complex (various cultivars)	ဒန်းဇင်း E: dan^{3} zin^{3}			dancing lady orchid	n
	ကရမက်ဒန်းဇင်းအဖြူ	kərəmeʔ dan^{3} zin^{3} əphyu^{2}	false.sandalwood-dancing-white		
	ကြယ်ဒန်းဇင်းညို	cɛ^{2} dan^{3} zin^{3} nyo^{2}	star-dancing-brown		
	ပနားမားဒန်းဇင်း	pəna^{3} ma^{3} dan^{3} zin^{3}	Panama-dancing		
	လိမ္မော်ရောင်ဒန်းဇင်း	lein^{2} mɔ^{2} yaun^{2} dan^{3} zin^{3}	orange-dancing		
Tolumnia sp.	ယင်ကောင်ဒန်းဇင်း	yin^{2} gaun^{2} dan^{3} zin^{3}	fly(insect)-dancing		n
?*Aliceara* Sunday Best, *Aliceara* Winter Wonderland, *Brassia maculata* R.Br.	ကြယ်ဒန်းဇင်း	cɛ^{2} dan^{3} zin^{3}	star-dancing		n
